# Screening the MayBridge Rule of 3 Fragment Library for Compounds That Interact with the *Trypanosoma brucei myo*-Inositol-3-Phosphate Synthase and/or Show Trypanocidal Activity

**DOI:** 10.4061/2011/389364

**Published:** 2011-05-17

**Authors:** Louise L. Major, Terry K. Smith

**Affiliations:** Biomolecular Science, The North Haugh, The University of St. Andrews, Fife, Scotland, KY16 9ST, UK

## Abstract

Inositol-3-phosphate synthase (INO1) has previously been genetically validated as a drug target against *Trypanosoma brucei*, the causative agent of African sleeping sickness. Chemical intervention of this essential enzyme could lead to new therapeutic agents. Unfortunately, no potent inhibitors of INO1 from any organism have been reported, so a screen for potential novel inhibitors of *T. brucei* INO1was undertaken. Detection of inhibition of *T. brucei* INO1 is problematic due to the nature of the reaction. Direct detection requires differentiation between glucose-6-phosphate and inositol-3-phosphate. Coupled enzyme assays could give false positives as potentially they could inhibit the coupling enzyme. Thus, an alternative approach of differential scanning fluorimetry to identify compounds that interact with *T. brucei* INO1 was employed to screen ~670 compounds from the MayBridge Rule of 3 Fragment Library. This approach identified 38 compounds, which significantly altered the T_m_ of TbINO1. Four compounds showed trypanocidal activity with ED50s in the tens of micromolar range, with 2 having a selectivity index in excess of 250. The trypanocidal and general cytotoxicity activities of all of the compounds in the library are also reported, with the best having ED50S of ~20 **μ**M against *T. brucei*.

## 1. Introduction

Human African Trypanosomiasis (HAT), also called African sleeping sickness, is caused by the extracellular protozoan parasite *Trypanosoma brucei.* HAT is a potentially fatal disease with ~200 000 new cases per year in sub-Saharan Africa [1]. Despite this, current drugs are often toxic and difficult to administer, highlighting the urgent need for new, more effective drug therapies. *T. brucei* is able to survive in the hosts' bloodstream due to a dense coat (5 × 10^6^ dimers/cell) of variant surface glycoprotein (VSG) [2, 3]. This coat acts as a diffusion barrier and enables the cell to avoid the hosts' innate immune system by a specialised process of antigenic variation, utilising a repertoire of more than 1000 different VSG genes [4, 5]. Although the VSG coat is systematically changed, it is always attached to the plasma membrane via a glycosylphosphatidylinositol (GPI) anchor [6, 7]. GPI anchors are ubiquitous to eukaryotes and comprise of the basic core structure of NH_2_CH_2_CH_2_PO_4_H-6Man*α*1-2Man*α*1-6Man*α*1-4GlcN*α*1-6$\underline{\text{D}}$-*myo*-inositol-1-HPO_4_-lipid (EtN-*P*-Man_3_GlcN-PI), with a lipid moiety of diacylglycerol, alkylacylglycerol, or ceramide [8]. Previously, the biosynthesis of GPI anchors in bloodstream form *T. brucei* has been both genetically and chemically validated as a therapeutic drug target [9–11].

The *de novo* synthesis of *myo-*inositol is a ubiquitous process occurring in almost all eukaryotes studied. It is the result of the concerted actions of two enzymes: firstly an $\underline{\text{D}}$-*myo*-inositol-3-phosphate synthase (INO1) which isomerases glucose-6-phosphate to $\underline{\text{D}}$-*myo*-inositol-3-phosphate and secondly, an inositol monophosphatase which dephosphorylates the $\underline{\text{D}}$-*myo*-inositol-3-phosphate to yield *myo-*inositol [12]. Previously, through the creation of a conditional knockout cell line of INO1, it was demonstrated that the *de novo* synthesis of *myo*-inositol is essential to the survival of bloodstream form *T. brucei* [13, 14]. Surprisingly, the deletion of INO1 could not be overcome by the inclusion of extra *myo*-inositol in the media, which is in striking contrast to all other INO1 null mutants described to date. Intriguingly, further analysis showed that there was no significant decrease in the level of *myo*-inositol in the conditional knockout cells grown under nonpermissive conditions, showing that the cells were not exhibiting the typical “inositol-less” death phenotype described for all other INO1 mutants [12, 15, 16]. *In vivo* labelling and localisation studies of INO1 [13, 14] and the *T. brucei* phosphatidylinositol synthase [17] suggested that the *de novo* synthesised *myo-*inositol is the primary source of *myo*-inositol used in the formation of phosphatidylinositol for GPI anchors and that there was a distinction or compartmentalisation of the *de novo* synthesised *myo*-inositol from that obtained from extracellular sources. The clear dependence by bloodstream form *T. brucei* on *de novo* synthesised *myo*-inositol for GPI anchor biosynthesis has not been described for any other organism to date and shows a unique avenue which could be exploited for future therapeutic drug design.

The MayBridge Rule of 3 Fragment Library (May Ro3) is a relatively small collection of chemical entries that are pharmacophore rich. The rule of 3 refers to compliance to the following criteria: MW ≤ 300, cLogP ≤ 3.0, H-Bond Acceptors ≤ 3, H-Bond Donors ≤ 3, Rotatable bonds (Flexibility Index) ≤ 3, and Polar Surface Area ≤ 60 Å [18]. The library has quantifiable diversity through the application of standard chemometrics, assured aqueous solubility to ≥1 mM using LogS and high purity (≥95%).

As *T. brucei* INO1 (TbINO1) is a genetically validated drug target and screening for inhibitors is problematic due to the nature of the reaction and/or the use of a coupled enzyme assay, differential scanning fluorimetry was employed to look for compounds that interact with TbINO1. Thus, ~670 compounds from the May Ro3 fragment library were screened and their trypanocidal and general cytotoxic activities determined.

## 2. Experimental

### 2.1. Materials

All materials unless stated were purchased either from Sigma/Aldrich or Invitrogen. Access to the Maybridge Rule of 3 (May Ro3) library, was kindly provided by Dr Rupert Russell (St Andrews). Stock solutions of the compounds (2 M) were prepared in DMSO and kept in master plates at 200 mM in DMSO (100%) by Dr Margaret Taylor (St Andrews). These were replated into daughter (working) plates occupying the central 80 wells of a 96-well plate, at 10 mM in 5% DMSO, allowing the two outside columns for positive and negative controls.

### 2.2. TbINO1 Protein Overexpression and Purification

Large-scale recombinant expression and purification of TbINO1 was conducted using the construct pET15b-TbINO1 in BL21 Rosetta (DE3) cells, and TbINO protein was purified by Ni affinity chromatography, eluted with 100 mM imidazole, 20 mM Tris pH 7.5, and 300 mM NaCl. The His-tagged protein was then dialysed against 20 mM Tris pH 7.5, 50 mM NaCl, 5 mM DTT and stored with 20% glycerol, at −80°C for up to 12 months without loss of activity. Full details of expression vector construction and purification will be published elsewhere (Martin, K. L. and T. K. Smith unpublished).

### 2.3. Differential Scanning Fluorimetry with TbINO1

Differential scanning fluorimetry was set up in 96-well PCR plates using a reaction volume of 100 *μ*L. Shifts in TbINO1 T_m_ with ligand binding were observed when ammonium acetate and NAD^+^ were present. Samples contained 2 *μ*M TbINO1, 2 mM Ammonium Acetate, 1 mM NAD^+^, 10 mM HEPES pH 7.5, 50 mM NaCl, and 1.25 working stock of Sypro Orange (Invitrogen, as a 5000 times stock). Compounds from the May Ro3 fragment library (1 mM) and positive controls, glucose-6-phosphate as substrate (5 mM) and 2-deoxy-glucose-6-phosphate as inhibitor (4 mM) were added as required.

Differential fluorimetric scans were performed in a real-time PCR machine (Stratagene Mx3005P with software MxPro v 4.01) using a temperature scan from 25°C to 95°C at 0.5°C min^−1^. Data were then exported to Excel for analysis using “DSF analysis” modified from the template provided by Niesen et al. [19]. T_m_ values were calculated by nonlinear regression, fitting the Boltzmann equation to the denaturation curves using GraFit.

### 2.4. Cytotoxicity Studies

The trypanocidal activity of all compounds (final 0.5 mM, 0.5% DMSO) against cultured bloodstream *T. brucei* (strain 427) was determined using the Alamar Blue viability test as described previously [20].

Cytotoxic effects against HeLa and A549 cells were determined in a similar manner. Briefly, the cells were cultured in DMEM supplemented with 10% FCS and 2 mM L-Glutamine. Cells were plated at initial cell concentration of 2 × 10^4^ cells/well and incubated with the compounds for ~65 hours prior to addition of Alamar Blue solution for a further 5 hours.

## 3. Results and Discussion

### 3.1. Is TbINO1 Amenable to Differential Scanning Fluorimetry? 

Inositol-3-phosphate synthase has previously been genetically validated as a drug target against *T. brucei* [13, 14], and is a prime candidate for chemical intervention as a therapeutic against African sleeping sickness. Unfortunately, no potent inhibitors of INO1 from any organism have been reported; therefore, it was decided to undertake a screen for potential novel inhibitors of TbINO1. Screening for inhibitors of TbINO1 is problematic due to the difficulty in following the reaction, that is, having to directly differentiate between glucose-6-phosphate and inositol-3-phosphate, or alternatively using a coupled enzyme assay, where a compound could potentially inhibit the coupled enzyme [14]. Thus, an alternative approach was taken, using differential scanning fluorimetry. Differential scanning fluorimetry has been used to identify compounds that interact with a protein, either to stabilise or destabilise it, and therefore influencing the protein's T_m_ (melting point) [21].

**Figure 1:**
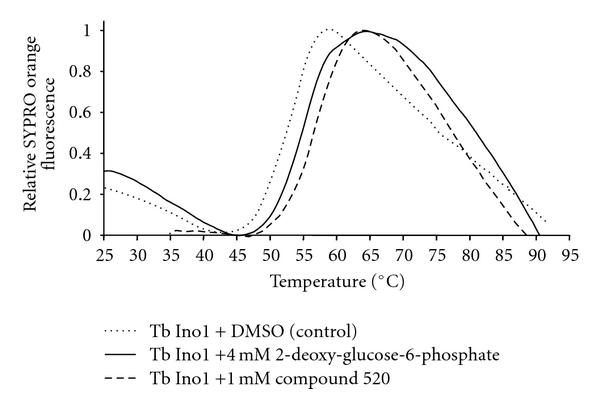
Typical differential fluorimetry scans of TbINO1. Differential fluorimetric scans were performed and analysed as described in Experimental. TbINO1 + DMSO (control) dotted line, TbINO1 + 4 mM 2-deoxy-glucose-6-phosphate (positive control) solid line, and TbINO1 + 1 mM compound 520, dashed line.

TbINO1 was subjected to differential scanning fluorimetry to ascertain if this approach was possible. The T_m_ for TbINO1 was determined in the presence of NAD^+^ and ammonium acetate, both known cofactors and stimuli of INO1 activity [13, 14, 22]; in a typical experiment, T_m_s were consistently ~51.4°C (4 samples, range 51.36–51.39°C) (Figure 1, dotted line). The substrate glucose-6-phosphate (5 mM) increased the T_m_ by 0.70 ± 0.28°C; however, 2-deoxy-glucose-6-phosphate (4 mM), a known inhibitor of INO1s [22–24], increased the T_m_ value by +2.84 ± 0.47°C (Figure 1, solid line). These encouraging results allowed validation of a screening program; thus, glucose-6-phosphate and 2-deoxy-glucose-6-phosphate were used as positive controls, and DMSO (in which all compounds were dissolved) as a negative control on all subsequent screening plates.

### 3.2. Screening of the May Ro3 Fragment Library for Compounds That Interact with Tbino1 by Differential Scanning Fluorimetry

As TbINO1 was amenable to screening by differential scanning fluorimetry, ~670 compounds from the May Ro3 fragment library were screened. Additionally, their trypanocidal and general cytotoxicity activities, against bloodstream *T. brucei* and HeLa and A549 cells were assessed (see Supplementary Table 1 in Supplementary Material available online at doi: 10.4061/2011/389364).

From this large amount of data, 38 compounds at 1 mM interacted with TbINO1 with a ΔT_m_ > +1.5°C (Table 1). Of these compound 520, 2-(2-furyl)benzoic acid, stabilised the protein the greatest with a ΔT_m_ + 4.29 ± 0.07°C (Figure 1, dashed line). It is interesting to note that the heterocyclic furan moiety is a common feature in several of the top hits (Table 1, compounds 520, 75, 30, 28, and 383).

Other similarities between the top hits are the presence of a carboxylic acid (Table 1, compounds 520, 75, 28, 513) or a methanol group (Table 1, compounds 186, 30, 383) attached to an aromatic ring. An obvious conclusion is that these moieties form important hydrogen bonds to the protein, in a similar orientation to each other with respect to the aromatic ring to which they are attached. 

The trypanocidal and cytotoxicity activities of these 38 compounds (Table 1) revealed that 9 of them killed more than 35% of *T. brucei* at 0.5 mM. These were investigated further, and their ED50s for both *T. brucei* and HeLa cells were determined (Table 1). Of these compound 162, 1H-indol-3-ylmethanol with a ΔT_m_ + 2.31 ± 0.06°C (Table 1), was the most potent with an ED50 of 31 ± 1.4 *μ*M, but was also cytotoxic against HeLa cells, ED50 of 103 ± 6 *μ*M, thus giving a very poor selectivity index of 3.3. However, another compound 256, 2-quinolinylmethanol, structurally very similar to compound 162, that has an ED50 of 40.1 ± 1.2 *μ*M, showed less cytotoxicity against HeLa cells, giving a selectivity index of ~21.

Interestingly, the top TbINO1 differential scanning fluorimetry hit, compound 520, also showed trypanocidal activity, ED50 of 76 ± 6 *μ*M, but no cytotoxicity towards HeLa cells at 20 mM, thus giving a selectivity index >256. However, the best selectivity index from these TbINO1 differential scanning fluorimetry hits (Table 1) was compound 239, 2-amino-4-methylthiophene-3-carboxamide, with a *T. brucei* ED50 of 63 ± 2.5 *μ*M and a selectivity index >317.

It is interesting to note that compound 520 contains a carboxylic acid and, therefore, should be impervious to membranes by passive diffusion; this seems to be true for the close analogous compound 513, 2-(1H-pyrrol-1-yl)benzoic acid, which also interacts with TbINO1 strongly but has no trypanocidal or general cytotoxicity activity at 0.5 mM (Table 1).

Puzzlingly, this suggests that some carboxylic acid containing compounds, such as 520, may be specifically and actively taken up by *T. brucei*, while other closely related carboxylic acid containing compounds are not.

The TbINO1 differential scanning fluorimetry hits will be investigated further in the future, as outlined in the conclusions.

### 3.3. Screening of the May Ro3 Fragment Library for Trypanocidal Compounds

The most potent trypanocidal compounds (ED50s < 100 *μ*M) of the ~670 compounds from the May Ro3 fragment library (Supplementary Table 1) were investigated further by determining their ED50s in both *T. brucei *and HeLa cells (Table 2). From first observations, it is clear that the vast majority of these compounds contain a primary amine, with the most potent compounds (ED50s < 40 *μ*M), 269, 270, and 348, containing an aromatic primary amine. Unfortunately, most of the primary amine containing compounds are cytotoxic and thus have poor selectivity indexes (0.5–21). The only exceptions are compounds 520 and 239 (both of which do not contain a primary amine), as discussed earlier, and showed no cytotoxicity at 20 mM (Tables 1 and 2) with selectivity indexes greater than 260. 

The structure activity relationship of the most potent trypanocidal compound from the library, compound 269 (4-chloro-5-fluorobenzene-1,2-diamine), with an ED50 of 20.1 ± 1.03 *μ*M, was investigated further. A series of analogues were purchased and tested in parallel; this included analogues that had the two amino groups, but with only one of the halides or one of the halides replaced by a methyl group or no halides. Other analogues maintained the halides, and reduce the amino groups to one, or by masking both free amines as a benzimidazole.

*T. brucei *and HeLa cell ED50s values were determined for this collection of compounds (Table 3).

Repurchased compound 269, gave ED50s similar to those obtained earlier (20.1 ± 1.03 and 31.1 ± 1.22 *μ*M). As to the structure activity relationship of the analogues, it is apparent that the two amino groups seem to be important for potent trypanocidal activity, as both compounds 269b and 269d have dramatically increased ED50s. 

The presence of two bulky groups opposite to the amine groups seems detrimental as shown by compound 240 (4-chloro-5-methylbenzene-1,2-diamine); a compound from the May Ro3 fragment library (Supplementary Table 1).

The absence of one or both of the halides does not alter the *T. brucei* ED50s dramatically, as demonstrated by compound 269a (benzene-1,2-diamine), with an ED50 of 35.2 ± 3.0 *μ*M (Table 3). The HeLa cell cytotoxicity of various benzene-1,2-diamine analogues varies dramatically, however the selectivity indexes are poor, the best being the original compound 269, 4-chloro-5-fluorobenzene-1,2-diamine, with a selectivity index of ~21 (Table 3)

Unfortunately, the benzene-1,2-diamine analogues are thought to be carcinogenic, as they are suspected to be able to interchelate DNA, although direct proof of this is still lacking [25]. These diamines are also well known to form Schiff bases and are often used for derivatisation of natural ketones and aldehydes, such as methglyoxal [26]. Ironically, it is also this Schiff base capability, which has led them to be investigated as possible anticancer, antibacterial, antifungal and antiviral agents [27, 28]. Either or both of the DNA interchelating, or Schiff base capabilities could be the possible mode of action for the trypanocidal activity. To avoid the obvious possible problems associated with carcinogenic compounds, while still maintaining the dual functionality that seems to be important for potency, compound 269e (2-amino-1-hydroxy-benzene or 2-amino-phenol), was purchased and tested. This proved to be the most potent trypanocidal agent tested in this study with an ED50 of 20.0 ± 0.3 *μ*M; however, it was cytotoxic to the HeLa cells (Table 3). Despite this, the relatively simple molecule (2-amino-phenol) has been shown to have anti-microbial activity [29, 30].

Considering these are very simple molecules, it is surprising that they are trypanocidal at low-micromolar concentrations, highlighting the importance of screening programs to identify novel pharmacophores.

## 4. Conclusions

In this work, screening of a comparatively small fragment library for thermal stabilisation of TbINO1 has allowed identification of several novel compounds that interact strongly and stabilise TbINO1. Unexpectedly, several of the significant hits are also trypanocidal with ED50 values in the 30–80 *μ*M range, despite being relative simple molecules. 

Several other compounds from the May Ro3 library showed low-micromolar trypanocidal activity. The majority of the most potent hits contain primary amines whose mode of action could be via Schiff-base formation, while some of the diamines could also be acting by inter-chelating DNA, thus interfering with cell-cycle progression/cell division.

Unfortunately, some of these compounds are cytotoxic against mammalian cells and thus are unlikely to progress as lead compounds. However, the biological activity of related compounds such as 269e (2-amino-phenol), also known to have anti-microbial activity, will be investigated further.

Future work outside the scope of this study will include the following:

- investigating if the lead compounds that interacted with TbINO1 inhibit its activity, in an *in vitro* coupled enzyme assay,
- investigating the mode of killing by the lead compounds that interacted with TbINO1 by undertaking various *in vivo* labelling experiments to ascertain if they are inhibiting TbINO1, thus causing a lack of *de novo* synthesised *myo*-inositol, required for PI synthesis for the essential GPI pathway,
- the direct interactions of compounds with TbINO1 will be investigated by protein crystallography studies.

## Supplementary Material

Supplementary Materials: The Material contains CAS numbers and structures of the Maybridge Rule of 3 fragment library, together with their activity in the TBIno1thermal shift assay, as well as their trypanocidal and cytotoxicity activity at 0.5 mM against bloodstream form *T. brucei* and mammalian cells (HeLA and A549), respectively.
Supplementary Table 2: Predicted values for all genes in different conditions.

## Figures and Tables

**Table 1:** Screening the MayBridge Rule of 3 Fragment Library for TbINO1 differential scanning fluorimetry hits with a Δ*T*_*m*_ > +1.5°C.

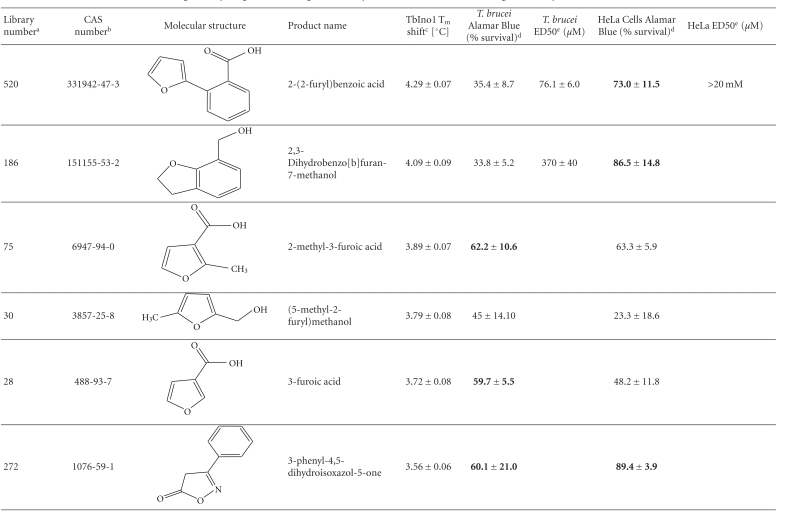 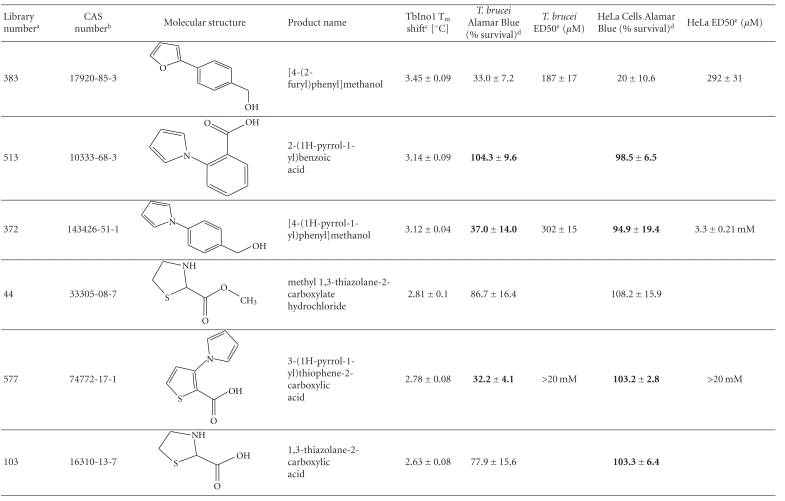 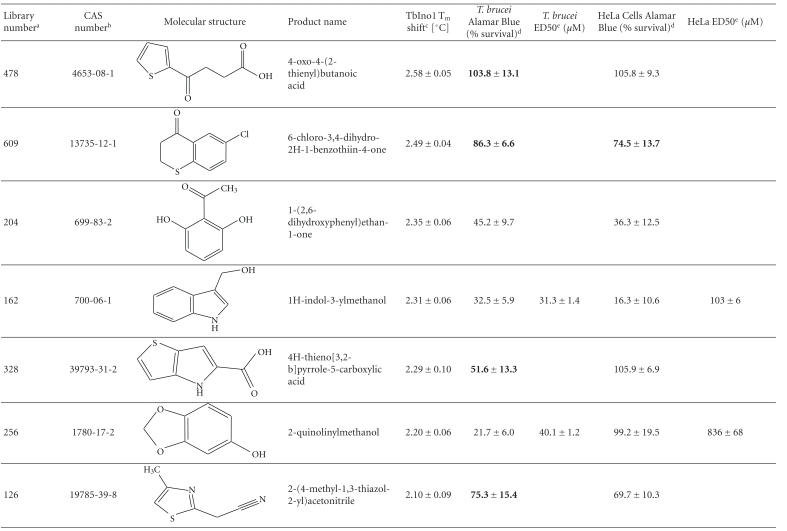 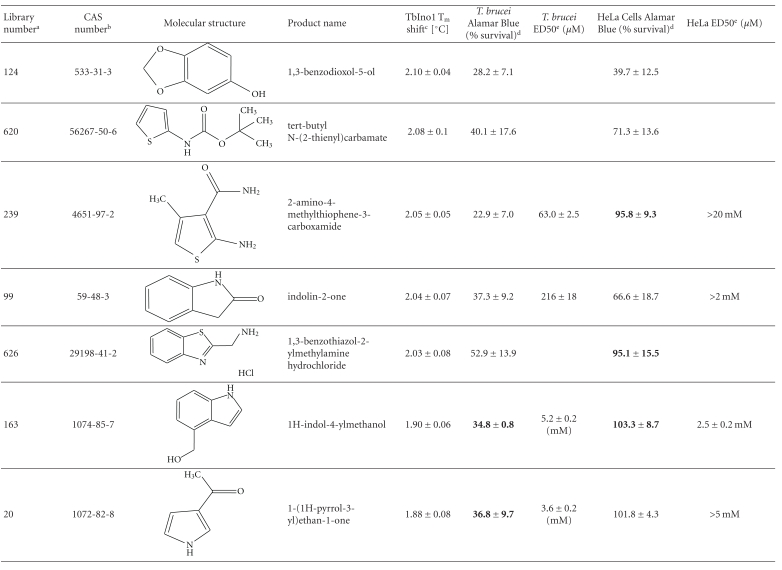 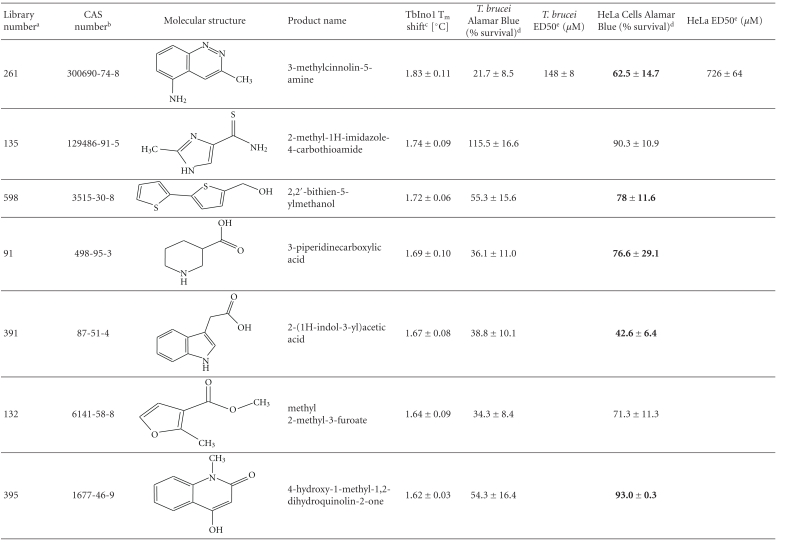 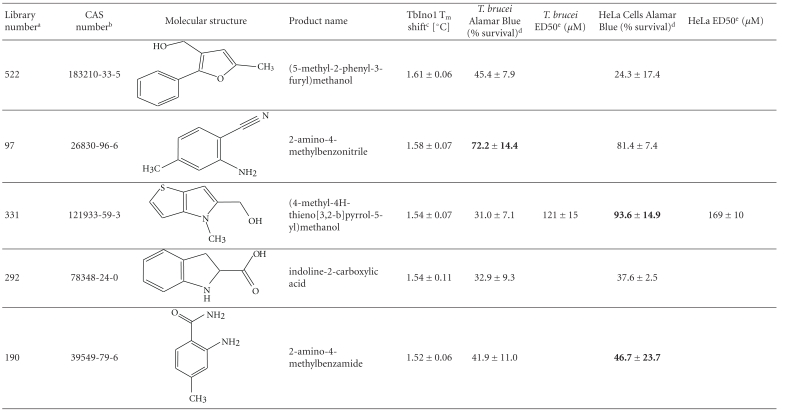

^a^Arbitrary Library number.

^b^CAS numbers are unique identifiers assigned by the “Chemical Abstracts Service” to describe every chemical described in the open access scientific literature.

^c^T_m_ shift in °C, observed for TbINO1 in the presence of compound (1 mM) and value is mean ± SD from the Boltzman curve fitting; see Section 2 for details, mean ± SD (*n* = 3).

^d^For cytotoxicity studies, see Section 2 for details and values are percentage of controls in the absence of compound, either mean ± SD (*n* = 3) or mean ± SE (*n* = 2), the latter being in bold.

^e^For trypanocidal and cytotoxicity activity, see Section 2 for details and values are ED50s ± SD (*n* = 4).

**Table 2:** Screening for trypanocidal compounds in the MayBridge Rule of 3 Fragment Library.

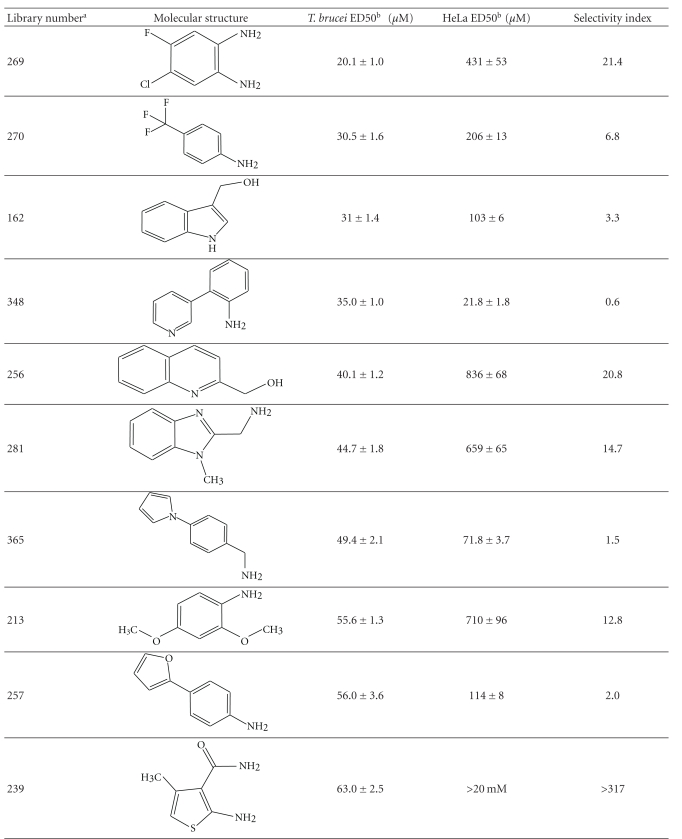 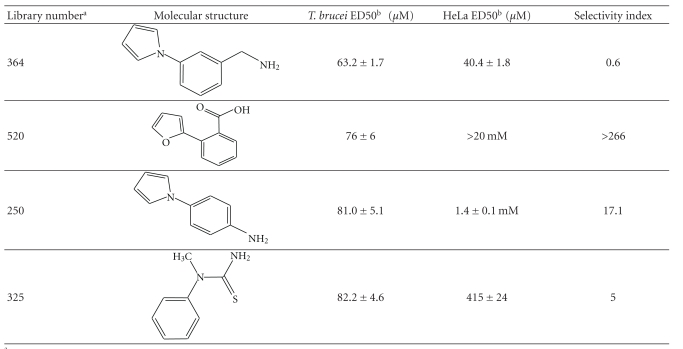

^a^Arbitrary Library number.

^b^For trypanocidal and cytotoxicity activity, see Section 2 for details and values are ED50s ± SD (*n* = 4).

^c^Selectivity index: *T. brucei* ED50/HeLa ED50.

**Table 3:** Structure activity relationship for trypanocidal activity of analogues of the diamine compound 269.

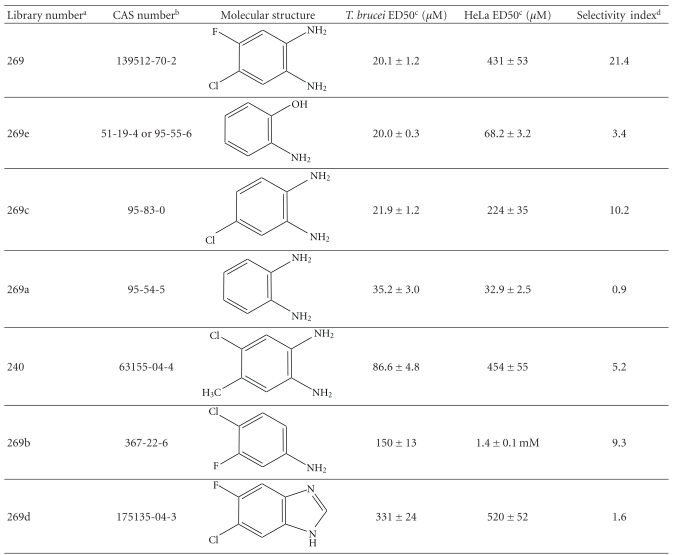

^a^Arbitrary Library number.

^b^CAS numbers are unique identifiers assigned by the “Chemical Abstracts Service” to describe every chemical described in the open access scientific literature.

^c^For trypanocidal and cytotoxicity activity, see Section 2 for details and values are ED50s ± SD (*n* = 4).

^d^Selectivity index: *T. brucei* ED50/HeLa ED50.
