# Supplementary material for: Screening the MayBridge Rule of 3 Fragment Library for Compounds That Interact with the Trypanosoma brucei myo-Inositol-3-Phosphate Synthase and/or Show Trypanocidal Activity
Source: Mol Biol Int. 2011 May 17;2011:389364. doi: 10.4061/2011/389364 (PMC3199943; doi:10.4061/2011/389364)
Supplement: Supplementary file 1 — Supplementary Materials: The Material contains CAS numbers and structures of the Maybridge Rule of 3 fragment library, together with their activity in the TBIno1thermal shift assay, as well as their trypanocidal and cytotoxicity activity at 0.5 mM against bloodstream form T. brucei and mammalian cells (HeLA and A549), respectively. Supplementary Table 2: Predicted values for all genes in different conditions. [file 389364.f1.pdf]

Supplementary Table 1: Screening the Maybridge Rule of Three Fragment Library: TbINO1 differential scanning fluorimetry and cytotoxicity studies against bloodstream form *T.brucei* , HeLa and A549 cells.

Library number[a]	CAS number[b]	Molecular Structure	Product Name	*Tb* Ino1 Tm Shift[c] [˚C]	*T. brucei* Alamar Blue[d]	HeLa Cells Alamar Blue[d]	A549 Cells Alamar Blue[e]
1	N/A	N/A	Control	-0.005 ± 0.152 f	99.5 ± 11	99.2 ± 10.8	100 ± 14
2	504-24-5	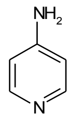	pyridin-4-amine	-0.22 ± 0.07	**98.8 ± 6.7**	102.7 ± 2.2	105.9
3	1530-88-7	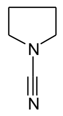	pyrrolidine-1-carbonitrile	-0.19 ± 0.06	82.5 ± 12.8	**88.5 ± 1.3**	114.9
4	300-87-8	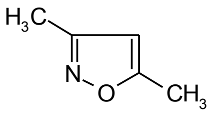	3,5-dimethylisoxazole	-0.4 ± 0.08	**116.6 ± 5.1**	107.7 ± 5.3	123.1
5	4543-47-9	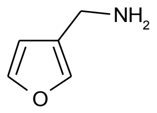	3-furylmethylamine	0.3 ± 0.06	**79.2 ± 7.9**	101.3 ± 5.3	112.7
6	31230-17-8	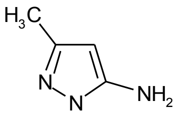	3-methyl-1H-pyrazol-5-amine	0.2 ± 0.06	94.9 ± 3.6	102.2 ± 3.3	121.4
7	1192-21-8	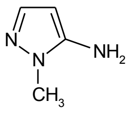	1-methyl-1H-pyrazol-5-ylamine	0.12 ± 0.06	96.4 ± 2.6	106.5 ± 6.2	123.6
8	1904-31-0	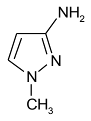	1-methyl-1H-pyrazol-3-amine	0.09 ± 0.07	93.4 ± 0.9	106.6 ± 7.8	122.1
9	1072-67-9	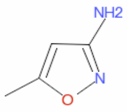	5-methylisoxazol-3-amine	0.13 ± 0.06	97 ± 14.3	**105 ± 6.2**	112.2
10	14678-02-5	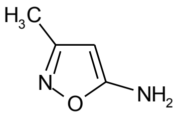	3-methylisoxazol-5-amine	-0.02 ± 0.07	67.7 ± 8.7	66 ± 4.6	87.2
11	3188-00-9	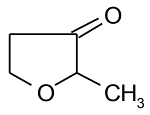	2-methyltetrahydrofuran-3-one	0.77 ± 0.06	**97.8 ± 15.4**	100.7 ± 5.4	85.3
12	29943-42-8	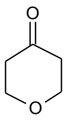	tetrahydro-4H-pyran-4-one	0.23 ± 0.07	103 ± 17.2	100.3 ± 3.5	97.4
13	505-66-8	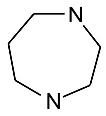	1,4-diazepane	0.4 ± 0.07	101.5 ± 15.4	88.5 ± 11.1	82.4
15	66121-66-2	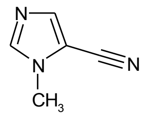	1-methyl-1H-imidazole-5-carbonitrile	0.08 ± 0.07	93.4 ± 4.5	100.6 ± 9.5	90
16	1603-41-4	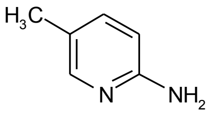	5-methylpyridin-2-amine	0.12 ± 0.06	66.2 ± 6.2	100.6 ± 1.1	89.4
17	1003-56-1	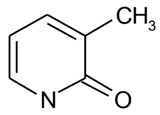	3-methyl-1,2-dihydropyridin-2-one	0.81 ± 0.08	**93.6 ± 17.8**	105.6 ± 11.4	87.2
18	586-95-8	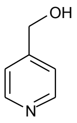	4-pyridinylmethanol	0.36 ± 0.07	**101.2 ± 1.3**	103.5 ± 3.9	102
19	3279-76-3	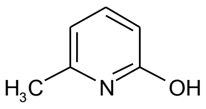	6-methylpyridin-2-ol	0.09 ± 0.08	94.5 ± 12.2	102.6 ± 7.6	85.6
20	1072-82-8	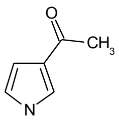	1-(1H-pyrrol-3-yl)ethan-1-one	1.88 ± 0.08	**36.8 ± 9.7**	101.8 ± 4.3	121.3

Supplementary Table 1: Screening the Maybridge Rule of Three Fragment Library: TbINO1 differential scanning fluorimetry and cytotoxicity studies against bloodstream form *T.brucei* , HeLa and A549 cells.

23	6705-33-5	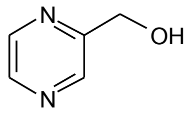	2-pyrazinylmethanol	0.27 ± 0.07	110.3 ± 11.8	77.5 ± 12.3	75.9
24	1072-91-9	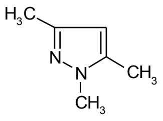	1,3,5-trimethyl-1H-pyrazole	-0.02 ± 0.07	**114 ± 12.5**	99.7 ± 11.5	88.5
25	931-36-2	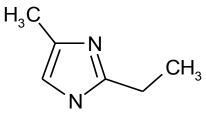	2-ethyl-4-methyl-1H-imidazole	-0.09 ± 0.06	89.6 ± 2.5	98 ± 7.8	83.9
26	3524-32-1	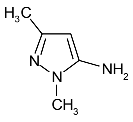	1,3-dimethyl-1H-pyrazol-5-amine	0.54 ± 0.07	**97.8 ± 4.7**	85.5 ± 9.4	70.1
27	88-14-2	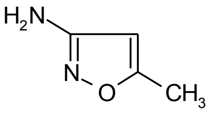	2-furoic acid	0.17 ± 0.06	**21.3 ± 1**	95.7 ± 9.4	106.6
28	488-93-7	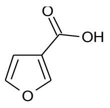	3-furoic acid	3.72 ± 0.08	**59.7 ± 5.5**	48.2 ± 11.8	111
29	371-41-5	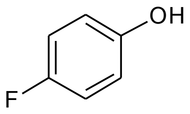	4-fluorophenol	0.23 ± 0.08	85.3 ± 12.3	96.2 ± 7.7	88.4
30	3857-25-8	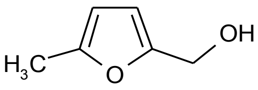	(5-methyl-2-furyl)methanol	3.79 ± 0.08	45 ± 14.1	23.3 ± 18.6	23.7
31	5157-08-4	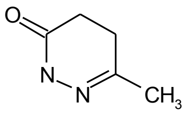	6-methyl-2,3,4,5-tetrahydropyridazin-3-one	0.22 ± 0.07	**110.1 ± 12.1**	85.4 ± 6.1	68.2
32	19947-75-2	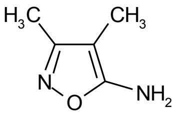	3,4-dimethylisoxazol-5-amine	-0.01 ± 0.07	96.2 ± 10.7	**76.4 ± 5.7**	71.7
33	154016-48-5	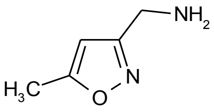	(5-methyl-3-isoxazolyl)methylamine	-0.07 ± 0.07	**75.7 ± 13.5**	64.3 ± 18.4	75.6
34	17334-08-6	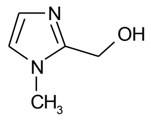	(1-methyl-1H-imidazol-2-yl)methanol	-0.16 ± 0.07	**133.4 ± 1.6**	97 ± 14	72.1
35	80-73-9	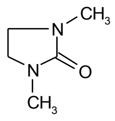	1,3-dimethylimidazolidin-2-one	0.04 ± 0.07	92.2 ± 13	101.1 ± 6.6	92.7
36	2815-34-1	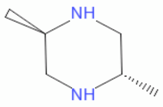	2,5-dimethylpiperazine	0.18 ± 0.08	81.1 ± 18.6	91.3 ± 12.1	76.6
37	344-25-2	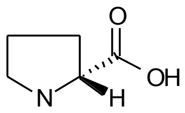	D-proline	0.41 ± 0.06	**102.9 ± 8.2**	94.8 ± 10.1	78.6
38	14542-12-2	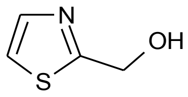	1,3-thiazol-2-ylmethanol	0.18 ± 0.09	**87.9 ± 9**	99.4 ± 4.1	95
39	108-33-8	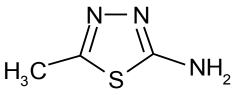	5-methyl-1,3,4-thiadiazol-2-amine	0.31 ± 0.08	102.8 ± 12	104.8 ± 14.5	72.3
40	6457-49-4	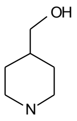	4-piperidylmethanol	-1.35 ± 0.1	**72.2 ± 8.1**	84.3 ± 9	104.2
42	16874-33-2	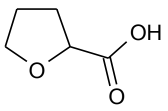	tetrahydro-2-furancarboxylic acid	0.3 ± 0.08	**107.5 ± 16.4**	81.9 ± 17	75.1
43	14774-37-9	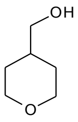	tetrahydro-2H-pyran-4-ylmethanol	-0.02 ± 0.08	112.7 ± 18.4	91.9 ± 5.9	79.2

Supplementary Table 1: Screening the Maybridge Rule of Three Fragment Library: TbINO1 differential scanning fluorimetry and cytotoxicity studies against bloodstream form *T.brucei* , HeLa and A549 cells.

44	120-72-9	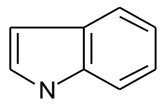	1H-indole	0.11 ± 0.06	28.4 ± 12	85.7 ± 19.3	75.7
45	21926-00-1	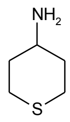	tetrahydrothiopyran-4-ylamine	0.24 ± 0.07	103.4 ± 13.8	88.6 ± 3.1	95.6
46	2739-97-1	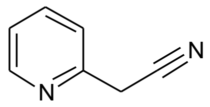	2-(2-pyridyl)acetonitrile	1.11 ± 0.24	44.1 ± 16.1	63.2 ± 17.9	82.6
47	29683-23-6	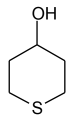	tetrahydro-2H-thiopyran-4-ol	-0.07 ± 0.07	**113.4 ± 7.5**	95 ± 10	74.9
48	17249-80-8	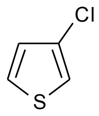	3-chlorothiophene	-0.2 ± 0.08	108.2 ± 17.8	95.5 ± 2	101
49	496-16-2	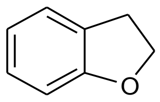	2,3-dihydrobenzo[b]furan	-1.08 ± 0.12	104.3 ± 18.1	102.1 ± 8.6	87.3
50	43036-06-2	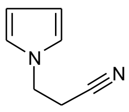	3-(1H-pyrrol-1-yl)propanenitrile	-0.67 ± 0.17	**90.7 ± 10.3**	**75.4 ± 6.4**	140.2
51	1194-02-1	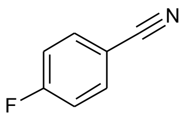	4-fluorobenzonitrile	0.1 ± 0.08	85.3 ± 5.2	90.9 ± 7.5	64.2
52	1122-69-6	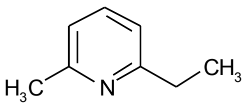	2-ethyl-6-methylpyridine	0.4 ± 0.08	80.8 ± 14	**89.7 ± 9.7**	70.8
53	31301-46-9	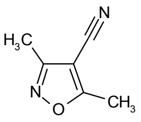	3,5-dimethyl-4-isoxazolecarbonitrile	0.52 ± 0.09	85 ± 13.8	89.3 ± 2.8	84.5
55	2687-25-4	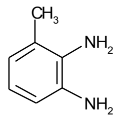	3-methylbenzene-1,2-diamine		30.7 ± 8.3	11.2 ± 7.2	26.5
56	6971-44-4	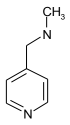	N-methyl-N-(4-pyridinylmethyl)amine	0.01 ± 0.08	**86.8 ± 7.8**	87.9 ± 9.9	78.5
57	20173-04-0	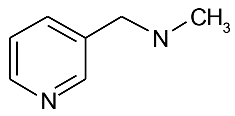	N-methyl-N-(3-pyridylmethyl)amine	-0.07 ± 0.07	**111.2 ± 6.8**	89.3 ± 9.6	79
58	98-98-6	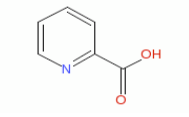	2-pyridinecarboxylic acid	-0.36 ± 0.08	**63 ± 15.4**	87.3 ± 6.1	97.9
60	55-22-1	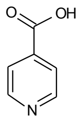	isonicotinic acid	-1.64 ± 0.13	90.7 ± 13.8	**98.3 ± 12.5**	117.5
61	536-90-3	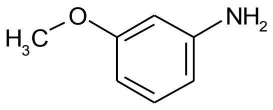	3-methoxyaniline	0.19 ± 0.07	**67.4 ± 6.7**	48.7 ± 8.9	69.2
62	1122-71-0	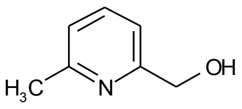	(6-methylpyridin-2-yl)methanol	-0.01 ± 0.07	95.7 ± 17.2	98.6 ± 7.3	67.5
63	6293-56-7	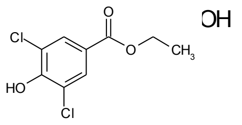	2-(3-pyridyl)ethan-1-ol	-0.07 ± 0.07	84.6 ± 9.4	104.8 ± 13.1	76.9
64	1122-43-6	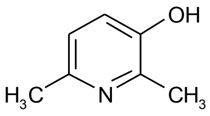	2,6-dimethylpyridin-3-ol	0.13 ± 0.07	**110.6 ± 6.3**	103.4 ± 13.2	80.6
65	20265-38-7	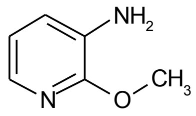	2-methoxypyridin-3-amine	1.46 ± 0.07	40 ± 11.5	**66.6 ± 18.7**	75.4

Supplementary Table 1: Screening the Maybridge Rule of Three Fragment Library: TbINO1 differential scanning fluorimetry and cytotoxicity studies against bloodstream form *T.brucei* , HeLa and A549 cells.

66	113293-71-3	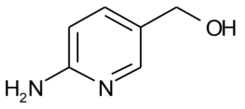	(6-amino-3-pyridinyl)methanol	0.24 ± 0.09	29.6 ± 6.1	80.3 ± 9.7	85.1
67	21917-76-0	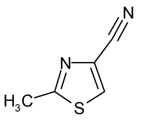	2-methyl-1,3-thiazole-4-carbonitrile	0.01 ± 0.07	106.5 ± 18	96.1 ± 9.2	89.7
68	4651-82-5	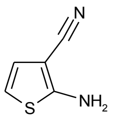	2-aminothiophene-3-carbonitrile		30.3 ± 11	**101.9 ± 3.1**	136.3
69	6973-60-0	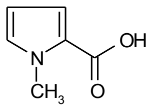	1-methyl-1H-pyrrole-2-carboxylic acid	-0.57 ± 0.08	99.1 ± 18.5	95.3 ± 2.5	70.2
70	100-82-3	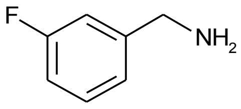	3-fluorobenzylamine	-1.88 ± 0.11	67.7 ± 18.9	104 ± 17.4	104.4
71	140-75-0	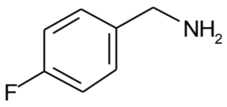	4-fluorobenzylamine	-0.17 ± 0.07	73.4 ± 16.8	93.3 ± 10.3	109.7
72	28466-21-9	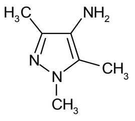	1,3,5-trimethyl-1H-pyrazol-4-amine	1.19 ± 0.08	20.8 ± 6.2	**78.5 ± 0.8**	70.9
73	499770-63-7	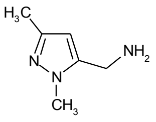	(1,3-dimethyl-1H-pyrazol-5-yl)methylamine	-0.08 ± 0.07	80 ± 15.5	**91.3 ± 0.6**	107.6
74	118-71-8	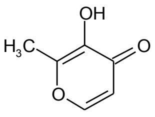	3-hydroxy-2-methyl-4H-pyran-4-one	1.13 ± 0.08	**44.5 ± 21.1**	106.7 ± 10.5	105.3
75	6947-94-0	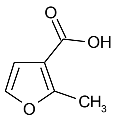	2-methyl-3-furoic acid	3.89 ± 0.07	**62.2 ± 10.6**	63.3 ± 5.9	110.4
76	153912-60-8	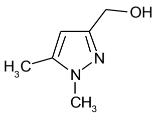	(1,5-dimethyl-1H-pyrazol-3-yl)methanol	0.56 ± 0.08	**85.4 ± 1.3**	**99.6 ± 4.5**	100.3
78	92901-94-5	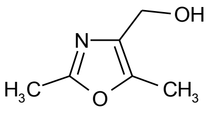	(2,5-dimethyl-1,3-oxazol-4-yl)methanol	0.13 ± 0.08	103.4 ± 10.5	92.7 ± 11.5	110.1
79	24295-03-2	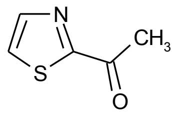	1-(1,3-thiazol-2-yl)ethan-1-one	-0.16 ± 0.07	113.9 ± 19.2	103.4 ± 16.6	94.5
80	5813-89-8	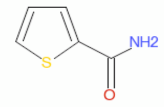	thiophene-2-carboxamide	-0.23 ± 0.07	99.6 ± 2.4	106.5 ± 17.5	90.3
81	30433-91-1	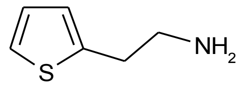	2-(2-thienyl)ethylamine	0.09 ± 0.07	**72.3 ± 9**	**116.8 ± 8.9**	119.2
82	17626-75-4	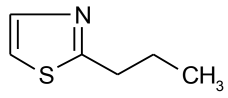	2-propyl-1,3-thiazole	0.22 ± 0.07	81.1 ± 11.9	96.8 ± 11	114.2
83	104163-35-1	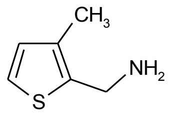	(3-methyl-2-thienyl)methylamine	0.43 ± 0.06	**95.7 ± 2.4**	95.4 ± 10	124.1
84	23775-39-5	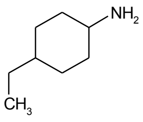	4-ethylcyclohexanamine	0.44 ± 0.06	**95.1 ± 12.8**	90.7 ± 14.2	118.3
85	527-72-0	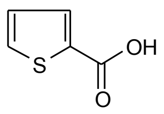	thiophene-2-carboxylic acid	0.24 ± 0.06	**101.1 ± 12.4**	100.2 ± 7.4	119
86	175334-72-2	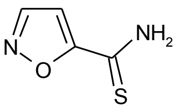	isoxazole-5-carbothioamide	0.94 ± 0.08	101.1 ± 3.2	101.9 ± 3.4	105.4

Supplementary Table 1: Screening the Maybridge Rule of Three Fragment Library: TbINO1 differential scanning fluorimetry and cytotoxicity studies against bloodstream form *T.brucei* , HeLa and A549 cells.

87	19214-08-5	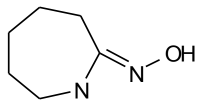	azepan-2-one oxime	0.52 ± 0.06	56.3 ± 9.9	92.3 ± 17.4	115
88	367-25-9	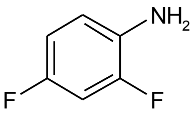	2,4-difluoroaniline	1.11 ± 0.07	**45.4 ± 8.9**	**87.6 ± 10.6**	132
89	372-39-4	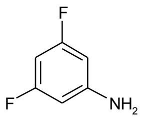	3,5-difluoroaniline	0.3 ± 0.07	**112.1 ± 10.7**	104.4 ± 10.5	132.4
90	498-94-2	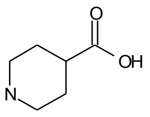	piperidine-4-carboxylic acid	0.5 ± 0.06	**75.1 ± 12.4**	106.3 ± 2	131.7
91	498-95-3	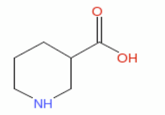	3-piperidinecarboxylic acid	1.69 ± 0.1	36.1 ± 11	**76.6 ± 29.1**	102.7
92	5337-03-1	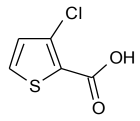	tetrahydro-2H-pyran-4-carboxylic acid	-0.35 ± 0.07	58.7 ± 14.3	78.4 ± 15.8	118.1
93	51673-83-7	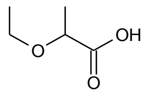	tetrahydropyran-2-carboxylic acid	0.55 ± 0.06	**67.2 ± 12**	**97.5 ± 2.4**	107.4
94	6575-13-9	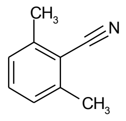	2,6-dimethylbenzonitrile	1.3 ± 0.06	86 ± 16.6	96.4 ± 13.9	114.4
95	933-67-5	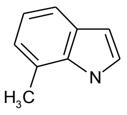	7-methyl-1H-indole	-4.86 ± 0.12	24.1 ± 5.2	11.9 ± 8.8	13.4
96	768-60-5	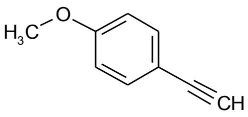	1-eth-1-ynyl-4-methoxybenzene	0.48 ± 0.06	104 ± 18.1	94 ± 4.9	111
97	26830-96-6	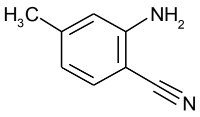	2-amino-4-methylbenzonitrile	1.58 ± 0.07	**72.2 ± 14.4**	81.4 ± 7.4	96.2
98	14191-95-8	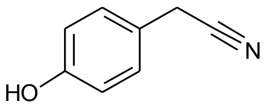	2-(4-hydroxyphenyl)acetonitrile	0.27 ± 0.05	**101.5 ± 0.9**	92.9 ± 6.5	114.9
99	59-48-3	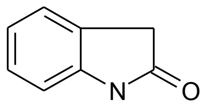	indolin-2-one	2.04 ± 0.07	37.3 ± 9.2	66.6 ± 18.7	228.9
100	58546-89-7	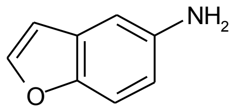	1-benzofuran-5-amine	0.78 ± 0.05	38 ± 6.4	80.2 ± 12.5	109.6
101	19335-11-6	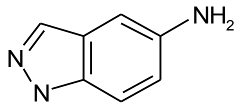	1H-indazol-5-amine		38.2 ± 11.6	31.7 ± 16.2	70.6
102	183428-90-2	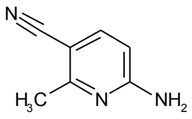	6-amino-2-methylnicotinonitrile	0.27 ± 0.06	68.4 ± 19.6	69.4 ± 12.4	76.1
103	16310-13-7	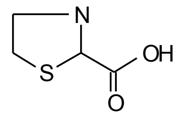	1,3-thiazolane-2-carboxylic acid	2.63 ± 0.08	77.9 ± 15.6	**103.3 ± 6.4**	107.3
104	2975-41-9	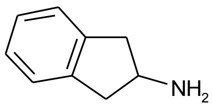	indan-2-amine	0.89 ± 0.05	28.9 ± 6.4	70 ± 18.5	122.9
107	36216-80-5	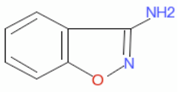	1,2-benzisoxazol-3-amine	0.95 ± 0.06	26.5 ± 5.8	76.9 ± 10.5	103.5
108	15871-85-9	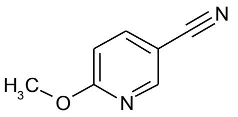	6-methoxynicotinonitrile	0.38 ± 0.07	**108.3 ± 14.1**	95 ± 10.2	125

Supplementary Table 1: Screening the Maybridge Rule of Three Fragment Library: TbINO1 differential scanning fluorimetry and cytotoxicity studies against bloodstream form *T.brucei* , HeLa and A549 cells.

109	4241-27-4	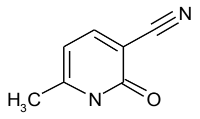	6-methyl-2-oxo-1,2-dihydropyridine-3-carbonitrile	0.22 ± 0.06	**108.6 ± 5.1**	97.4 ± 2	118.7
110	493-05-0	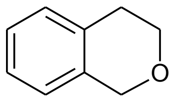	3,4-dihydro-1H-benzo[c]pyran	0.34 ± 0.06	**109.8 ± 0.6**	88.7 ± 19.2	114.3
111	34413-35-9	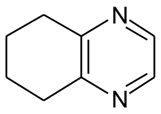	5,6,7,8-tetrahydroquinoxaline	0.16 ± 0.06	**109.7 ± 17.6**	**95.6 ± 0.8**	96.1
112	21725-69-9	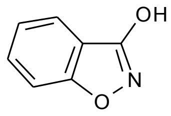	benzo[d]isoxazol-3-ol	0.23 ± 0.06	**100.2 ± 3**	83 ± 11.2	76.7
113	51586-20-0	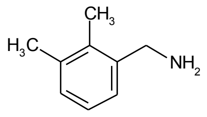	2,3-dimethylbenzylamine	0.12 ± 0.07	**74.5 ± 13.2**	89.3 ± 15.9	83.6
114	20154-03-4	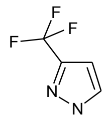	3-(trifluoromethyl)-1H-pyrazole	-0.07 ± 0.07	86.2 ± 19.7	85.9 ± 6.1	73.8
115	77326-36-4	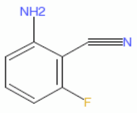	2-amino-6-fluorobenzonitrile	0.17 ± 0.07	**90 ± 9.9**	**71.3 ± 0.6**	77.4
116	7145-99-5	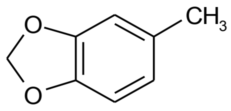	5-methyl-1,3-benzodioxole	0.38 ± 0.06	**114.9 ± 5.4**	82.7 ± 15.6	109.4
117	23787-80-6	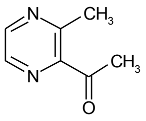	1-(3-methylpyrazin-2-yl)ethan-1-one	0.4 ± 0.06	**105.4 ± 17.6**	87.6 ± 11.3	88.3
118	20348-23-6	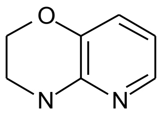	3,4-dihydro-2H-pyrido[3,2-b][1,4]oxazine	0.46 ± 0.06	**75.6 ± 3.7**	84.1 ± 18	92.3
119	6966-10-5	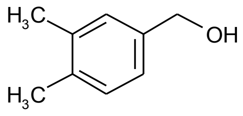	(3,4-dimethylphenyl)methanol	-0.28 ± 0.07	**102.7 ± 2.8**	84.7 ± 15.3	123.5
120	82380-18-5	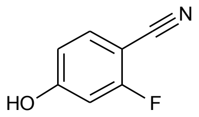	2-fluoro-4-hydroxybenzonitrile	0.41 ± 0.06	39 ± 9.1	18.6 ± 10.7	72.6
121	14268-66-7	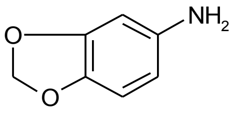	1,3-benzodioxol-5-amine	0.72 ± 0.07	42.4 ± 11.3	76.7 ± 16	109.9
122	1758-46-9	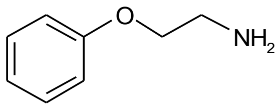	2-phenoxyethylamine	-0.03 ± 0.06	**64.6 ± 8.2**	73.6 ± 5.4	100.4
123	2227-79-4	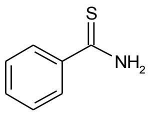	benzene-1-carbothioamide	0.42 ± 0.06	107.2 ± 16.2	89 ± 10.9	87.9
124	533-31-3	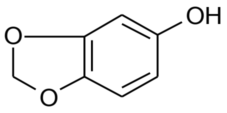	1,3-benzodioxol-5-ol	2.1 ± 0.04	28.2 ± 7.1	39.7 ± 12.5	53.6
125	455-36-7	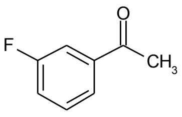	1-(3-fluorophenyl)ethan-1-one	-0.02 ± 0.07	**105.3 ± 17.2**	83 ± 14.8	101.3
126	19785-39-8	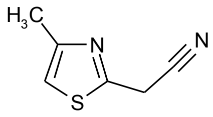	2-(4-methyl-1,3-thiazol-2-yl)acetonitrile	2.1 ± 0.09	**75.3 ± 15.4**	69.7 ± 10.3	98.4
128	352018-93-0	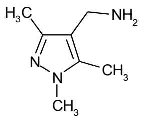	(1,3,5-trimethyl-1H-pyrazol-4-yl)methylamine	0.22 ± 0.06	**87 ± 9.9**	**80.3 ± 7**	87.7
129	445-29-4	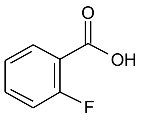	2-fluorobenzoic acid	0.13 ± 0.07	**123.5 ± 7.6**	78.6 ± 14.2	126.3

Supplementary Table 1: Screening the Maybridge Rule of Three Fragment Library: TbINO1 differential scanning fluorimetry and cytotoxicity studies against bloodstream form *T.brucei* , HeLa and A549 cells.

130	636-44-2	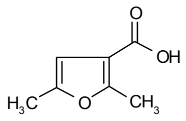	2,5-dimethyl-3-furoic acid	0.62 ± 0.08	**98.1 ± 6.8**	**84.7 ± 0.7**	120.6
131	935-13-7	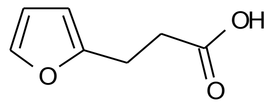	3-(2-furyl)propanoic acid	1.22 ± 0.09	52.6 ± 15.5	**69.8 ± 21.4**	98.1
132	6141-58-8	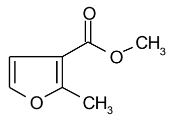	methyl 2-methyl-3-furoate	1.64 ± 0.09	34.3 ± 8.4	71.3 ± 11.3	100.4
133	31968-33-9	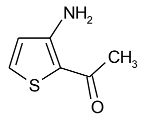	1-(3-amino-2-thienyl)ethan-1-one	1.24 ± 0.07	**56.3 ± 21.4**	83 ± 12.2	100.4
134	83933-16-8	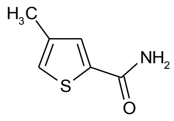	4-methylthiophene-2-carboxamide	0.61 ± 0.07	**73.7 ± 20.6**	83.1 ± 5.6	123.3
135	129486-91-5	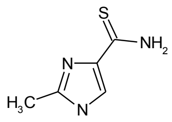	2-methyl-1H-imidazole-4-carbothioamide	1.74 ± 0.09	115.5 ± 16.6	90.3 ± 10.9	101.5
136	89-97-4	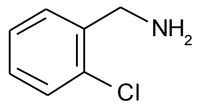	2-chlorobenzylamine	0.02 ± 0.07	**113.2 ± 5.8**	53.3 ± 12.2	90.4
138	5380-42-7	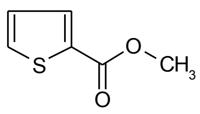	methyl thiophene-2-carboxylate	0.31 ± 0.05	**110.9 ± 2.8**	**89.4 ± 9.3**	120.9
139	14282-78-1	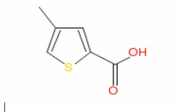	4-methylthiophene-2-carboxylic acid	0.08 ± 0.07	**121.9 ± 1.1**	**70.9 ± 10.7**	120.9
140	77358-26-0	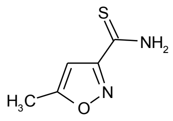	5-methylisoxazole-3-carbothioamide	0.2 ± 0.07	99.2 ± 19.8	89.2 ± 6.5	110.2
141	165115-15-1	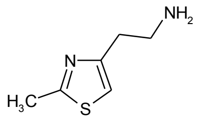	2-(2-methyl-1,3-thiazol-4-yl)ethylamine	-0.02 ± 0.08	**82.8 ± 0.9**	98.9 ± 7.6	104.6
142	34155-39-0	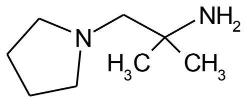	2-methyl-1-(1-pyrrolidinyl)-2-propanamine	0.13 ± 0.07	**70.6 ± 6.5**	89.9 ± 8.9	96.3
143	17849-38-6	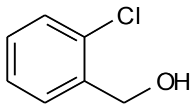	(2-chlorophenyl)methanol	0.46 ± 0.05	88.5 ± 19.7	93.3 ± 6.6	108.7
144	138564-16-6	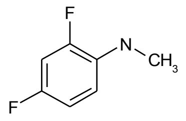	N1-methyl-2,4-difluoroaniline	1.09 ± 0.06	**38.1 ± 11.4**	79.3 ± 10.3	109.6
145	20485-41-0	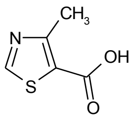	4-Methyl-thiazole-5-carboxylic acid	0.01 ± 0.07	**115.2 ± 9.7**	90.6 ± 16.1	114.5
146	35272-15-2	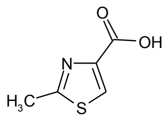	2-methyl-1,3-thiazole-4-carboxylic acid	0.1 ± 0.08	**102.5 ± 14.9**	87 ± 6.4	109
147	50382-32-6	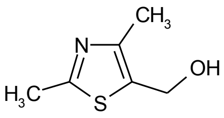	(2,4-dimethyl-1,3-thiazol-5-yl)methanol	0.14 ± 0.08	98 ± 16.2	93.6 ± 4.3	118.5
148	24044-76-6	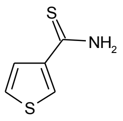	thiophene-3-carbothioamide	0.78 ± 0.08	74.6 ± 6.6	92.3 ± 7	99.6
149	670-95-1	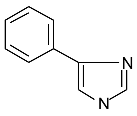	4-phenyl-1H-imidazole	0.24 ± 0.06	**86.3 ± 13.6**	80.2 ± 11.7	99.1
150	2458-26-6	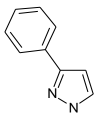	3-phenyl-1H-pyrazole	-0.45 ± 0.06	51.9 ± 12.6	74.6 ± 17.7	82

Supplementary Table 1: Screening the Maybridge Rule of Three Fragment Library: TbINO1 differential scanning fluorimetry and cytotoxicity studies against bloodstream form *T.brucei* , HeLa and A549 cells.

151	72692-99-0	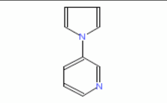	3-(1H-pyrrol-1-yl)pyridine	0.43 ± 0.06	**56.6 ± 19.2**	90.3 ± 6	90.1
152	580-22-3	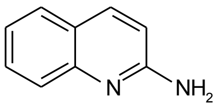	quinolin-2-amine	-0.43 ± 0.08	33.4 ± 6.7	38.2 ± 3	56.9
153	25475-67-6	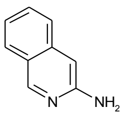	isoquinolin-3-amine	0.27 ± 0.06	**44.8 ± 5**	**88.5 ± 16.5**	117.6
154	14675-44-6	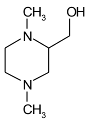	(1,4-dimethyl-2-piperazinyl)methanol	-0.04 ± 0.07	89.2 ± 16.1	96.9 ± 6.4	117.2
155	1443-80-7	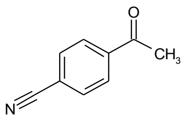	4-acetylbenzonitrile	0.19 ± 0.06	**99.3 ± 17.3**	87.3 ± 16.6	130.8
156	1006-68-4	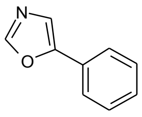	5-phenyl-1,3-oxazole	0.95 ± 0.06	35.1 ± 3.8	**77.5 ± 18.3**	104.2
157	17496-14-9	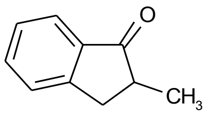	2-methylindan-1-one	0.14 ± 0.07	**108.6 ± 10.3**	94.7 ± 3.5	125.5
158	102308-97-4	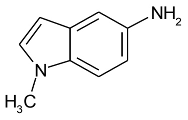	1-methyl-1H-indol-5-amine	-0.82 ± 0.1	42.7 ± 9.7	12.5 ± 9.1	59.3
159	14294-10-1	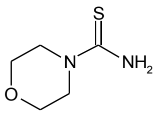	morpholine-4-carbothioamide	0.21 ± 0.05	113.3 ± 16.8	93.8 ± 9.3	108.6
160	2613-23-2	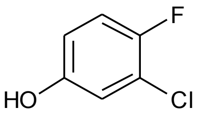	3-chloro-4-fluorophenol	0.02 ± 0.05	50.8 ± 16.5	94.9 ± 15.3	77.4
161	104-47-2	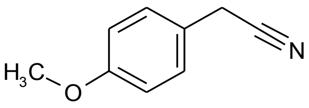	2-(4-methoxyphenyl)acetonitrile	-0.04 ± 0.08	100.9 ± 1.7	107.7 ± 3.1	88.7
162	700-06-1	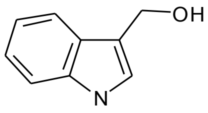	1H-indol-3-ylmethanol	2.31 ± 0.06	32.5 ± 5.9	16.3 ± 10.6	41.6
163	1074-85-7	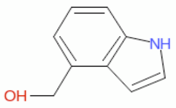	1H-indol-4-ylmethanol	1.9 ± 0.06	**34.8 ± 0.8**	**103.3 ± 8.7**	47
164	58537-99-8	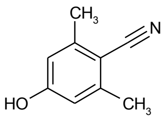	4-hydroxy-2,6-dimethylbenzonitrile	0.26 ± 0.08	84.7 ± 2.4	55.8 ± 12.1	90
165	37798-08-6	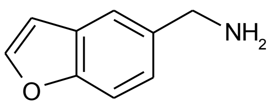	1-benzofuran-5-ylmethylamine	-0.26 ± 0.07	**81.5 ± 6.3**	**92.6 ± 8.6**	90.7
166	52601-70-4	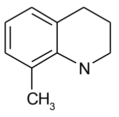	8-methyl-1,2,3,4-tetrahydroquinoline	0.06 ± 0.07	**55.8 ± 21.7**	**95.5 ± 1.6**	76
167	55038-01-2	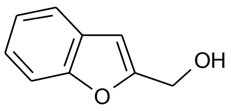	1-benzofuran-2-ylmethanol	-0.24 ± 0.09	**89.8 ± 4.7**	**84.9 ± 19.3**	78.2
168	31823-05-9	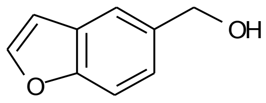	1-benzofuran-5-ylmethanol	0.39 ± 0.08	34.7 ± 8	**119.6 ± 11.4**	75.1
169	4856-97-7	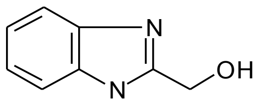	1H-benzimidazol-2-ylmethanol	0.04 ± 0.07	86.5 ± 2.7	**121.3 ± 15**	81.7
170	106429-29-2	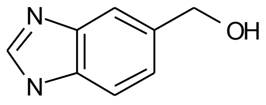	1H-benzimidazol-5-ylmethanol	0.15 ± 0.07	98.8 ± 1.6	**98.3 ± 4.2**	92

Supplementary Table 1: Screening the Maybridge Rule of Three Fragment Library: TbINO1 differential scanning fluorimetry and cytotoxicity studies against bloodstream form *T.brucei* , HeLa and A549 cells.

171	132213-07-1	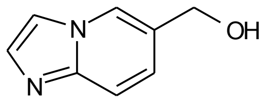	imidazo[1,2-a]pyridin-6-ylmethanol	0.84 ± 0.09	**81.7 ± 2.5**	94.1 ± 15.6	99.4
172	82090-52-6	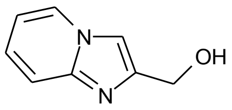	imidazo[1,2-a]pyridin-2-ylmethanol	0.44 ± 0.08	47.6 ± 14.2	**86.6 ± 15.1**	97.5
173	16400-13-8	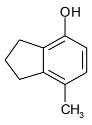	7-methylindan-4-ol	-0.53 ± 0.05	29.6 ± 6	**43.2 ± 15.6**	53.7
174	22699-70-3	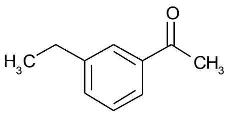	1-(3-ethylphenyl)ethan-1-one	0.8 ± 0.05	106.9 ± 13.3	100.3 ± 7.3	79.2
175	57319-65-0	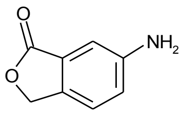	6-amino-1,3-dihydroisobenzofuran-1-one	0.04 ± 0.08	91.7 ± 12.2	105.9 ± 17	79.5
176	5580-34-7	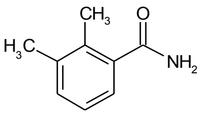	2,3-dimethylbenzamide	0.06 ± 0.07	106.6 ± 12.1	**88.2 ± 12.4**	68.7
177	5580-33-6	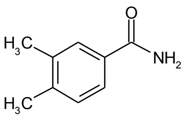	3,4-dimethylbenzamide	-0.28 ± 0.08	29.9 ± 7.7	81.7 ± 16.8	64.2
178	769-92-6	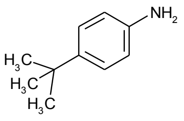	4-(tert-butyl)aniline	-0.21 ± 0.06	**101.1 ± 6**	**108.9 ± 18.8**	87.6
179	5369-19-7	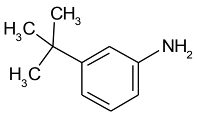	3-(tert-butyl)aniline	-0.37 ± 0.06	**92.3 ± 14.3**	99.3 ± 14.7	77.9
180	568577-84-4	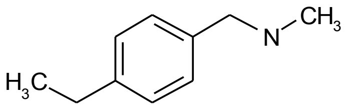	N-(4-ethylbenzyl)-N-methylamine	0.14 ± 0.08	101.8 ± 4.4	**99.8 ± 1.1**	87
181	154471-65-5	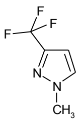	1-methyl-3-(trifluoromethyl)-1H-pyrazole	0.11 ± 0.08	105.2 ± 8	114.9 ± 9.9	58.4
182	6272-26-0	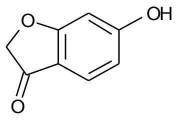	6-hydroxy-2,3-dihydrobenzo[b]furan-3-one	0.92 ± 0.09	56.2 ± 7.7	**119.6 ± 2.4**	65.6
183	89-71-4	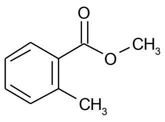	methyl 2-methylbenzoate	0.14 ± 0.08	99 ± 14.4	**114.2 ± 4.3**	51
184	1450-72-2	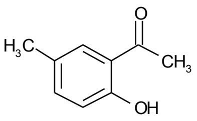	1-(2-hydroxy-5-methylphenyl)ethan-1-one	0.14 ± 0	**96.1 ± 4.8**	91.7 ± 11.6	53.1
185	99-36-5	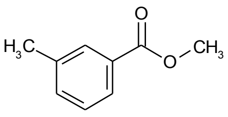	methyl 3-methylbenzoate	0.14 ± 0.09	110.5 ± 12.7	105.5 ± 10.4	61.3
186	151155-53-2	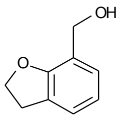	2,3-Dihydrobenzo[b]furan-7-methanol	4.09 ± 0.09	33.8 ± 5.2	**86.5 ± 14.8**	41.8
187	66158-96-1	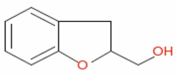	2,3-dihydro-1-benzofuran-2-ylmethanol	-0.04 ± 0.08	**66.8 ± 21.8**	94.3 ± 13.3	50.5
188	22977-34-0	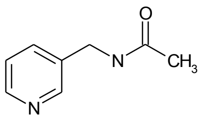	N1-(3-pyridylmethyl)acetamide	0.05 ± 0.07	88.2 ± 7	104.2 ± 7.3	69.5
189	6788-68-7	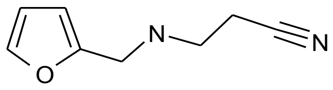	3-[(2-furylmethyl)amino]propanenitrile	0.75 ± 0.08	**81.5 ± 19.2**	92.5 ± 7.2	84.4
190	39549-79-6	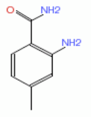	2-amino-4-methylbenzamide	1.52 ± 0.06	41.9 ± 11	**46.7 ± 23.7**	106.3

Supplementary Table 1: Screening the Maybridge Rule of Three Fragment Library: TbINO1 differential scanning fluorimetry and cytotoxicity studies against bloodstream form *T.brucei* , HeLa and A549 cells.

191	533-30-2	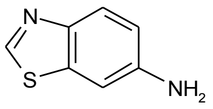	1,3-benzothiazol-6-amine	0.44 ± 0.11	47.7 ± 13.8	76.3 ± 15.1	38
192	17194-82-0	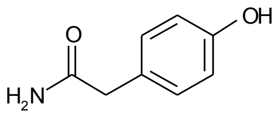	2-(4-hydroxyphenyl)acetamide	-0.02 ± 0.08	**107.7 ± 16.5**	96.9 ± 9.5	44.6
193	22013-33-8	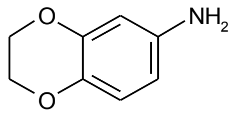	2,3-dihydro-1,4-benzodioxin-6-amine	0.4 ± 0.09	29.5 ± 5.5	**79.9 ± 13.6**	44.7
194	4518-10-9	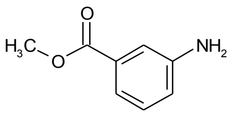	methyl 3-aminobenzoate	0.78 ± 0.06	89.8 ± 7	88.3 ± 9.2	38.9
195	22791-64-6	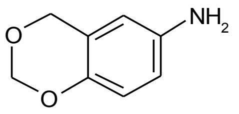	4,5-dihydro-1,3-benzodioxine-6-amine	1.16 ± 0.06	41.8 ± 8.8	**77.4 ± 10.5**	54.1
196	2305-36-4	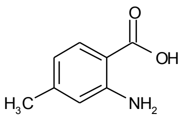	2-amino-4-methylbenzoic acid	1.1 ± 0.06	87.4 ± 19.7	**97.4 ± 4.1**	36.7
198	88653-55-8	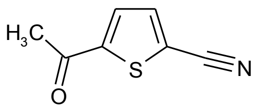	5-acetylthiophene-2-carbonitrile	0.24 ± 0.07	79.6 ± 18.5	93.3 ± 15.3	61.2
199	702-24-9	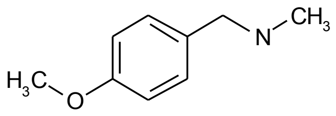	N-(4-methoxybenzyl)-N-methylamine	-0.11 ± 0.08	92.2 ± 16.8	95.1 ± 15.3	71
200	495-76-1	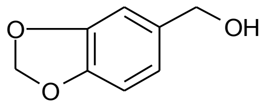	1,3-benzodioxol-5-ylmethanol	-0.07 ± 0.08	**84.2 ± 5.9**	85.2 ± 9.3	92.1
201	614-75-5	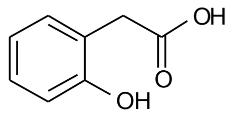	2-(2-hydroxyphenyl)acetic acid	0.65 ± 0.08	87.4 ± 8.7	94.1 ± 15.7	94.2
202	90-64-2	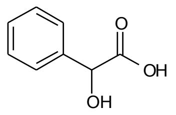	2-hydroxy-2-phenylacetic acid	0 ± 0.08	107.2 ± 16	96.7 ± 12.5	55.6
203	769-30-2	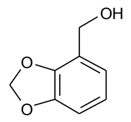	1,3-benzodioxol-4-ylmethanol	0.06 ± 0.08	74.8 ± 13.4	94.6 ± 14.1	60.8
204	699-83-2	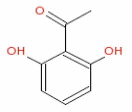	1-(2,6-dihydroxyphenyl)ethan-1-one	2.35 ± 0.06	45.2 ± 9.7	36.3 ± 12.5	34.4
205	825-03-6	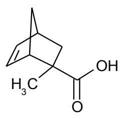	2-methylbicyclo[2.2.1]hept-5-ene-2-carboxylic acid	0.08 ± 0.08	**91.3 ± 1.8**	92.1 ± 17.2	53.8
206	16473-11-3	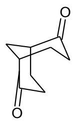	bicyclo[3.3.1]nonane-2,6-dione	-0.11 ± 0.08	93.2 ± 7.8	80.8 ± 7.6	42.1
207	25503-91-7	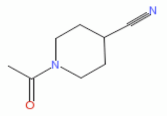	1-acetylpiperidine-4-carbonitrile	0.03 ± 0.07	96.5 ± 13.9	90 ± 2.9	65.3
208	6238-30-8	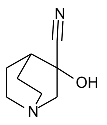	3-hydroxyquinuclidine-3-carbonitrile	-0.09 ± 0.07	101.6 ± 18	99.4 ± 11.2	61.8
209	768-95-6	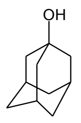	adamantan-1-ol	-0.12 ± 0.07	101.4 ± 11.5	92.2 ± 6.7	76.1
210	6575-11-7	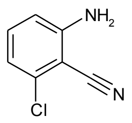	2-amino-6-chlorobenzonitrile	0.15 ± 0.08	93.5 ± 7.9	91.5 ± 10.8	89.2
211	3172-52-9	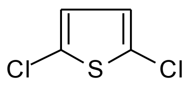	2,5-dichlorothiophene	0.02 ± 0.08	101.5 ± 10.9	99.6 ± 4.1	117.2

Supplementary Table 1: Screening the Maybridge Rule of Three Fragment Library: TbINO1 differential scanning fluorimetry and cytotoxicity studies against bloodstream form *T.brucei*, HeLa and A549 cells.

212	399-31-5	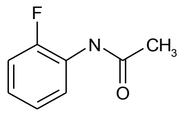	N1-(2-fluorophenyl)acetamide	-0.16 ± 0.08	97.3 ± 9.6	80.6 ± 6.6	65.4
213	2735-04-8	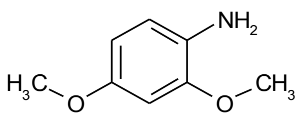	2,4-dimethoxyaniline		23.7 ± 5.8	14 ± 8.2	61.2
214	69687-80-5	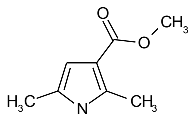	methyl 2,5-dimethyl-1H-pyrrole-3-carboxylate	0.78 ± 0.06	35.1 ± 4.4	**74.8 ± 18**	64.4
215	164670-44-4	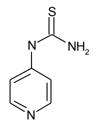	N-(4-pyridyl)thiourea	0.03 ± 0.08	118.6 ± 17	98.3 ± 16.5	59.6
216	118430-73-2	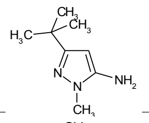	3-(tert-butyl)-1-methyl-1H-pyrazol-5-amine	0.15 ± 0.07	**97.5 ± 10.1**	80.1 ± 11.3	56.7
217	56004-83-2	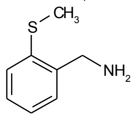	2-(methylthio)benzylamine	-0.07 ± 0.07	**81.9 ± 10.4**	**27.1 ± 0.5**	70.3
218	13589-72-5	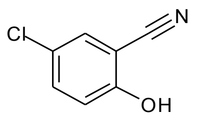	5-chloro-2-hydroxybenzonitrile	0.43 ± 0.07	**92.7 ± 13.6**	92 ± 11.8	67.7
219	405-50-5	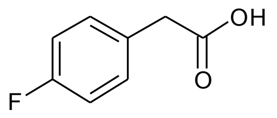	2-(4-fluorophenyl)acetic acid	-1.9 ± 0.61	101.2 ± 18	94.3 ± 13.4	98.4
220	394-35-4	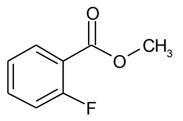	methyl 2-fluorobenzoate	-0.03 ± 0.1	108.4 ± 10.5	107.6 ± 17.6	99.5
221	349480-74-6	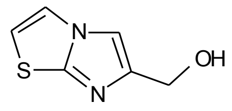	imidazo[2,1-b]thiazol-6-ylmethanol	0.09 ± 0.23	97.7 ± 7	106.6 ± 7.6	99.5
222	5122-77-0	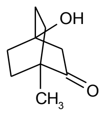	4-hydroxy-1-methylbicyclo[2.2.2]octan-2-one	-0.11 ± 0.09	106.5 ± 8.6	101.8 ± 5.9	82.3
223	68283-19-2	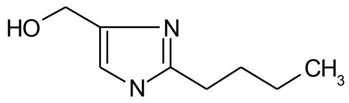	(2-butyl-1H-imidazol-4-yl)methanol	-0.17 ± 0.14	100.4 ± 6.7	105.2 ± 13.7	139.3
224	90322-18-2	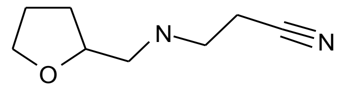	3-[(tetrahydrofuran-2-ylmethyl)amino]propanenitrile	0.21 ± 0.09	103.3 ± 15.4	96.5 ± 13.7	60
225	19155-86-3	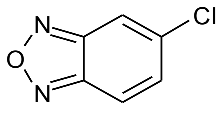	5-chloro-2,1,3-benzoxadiazole	0.81 ± 0.19	95 ± 12.2	91.4 ± 15.1	68.9
226	38205-60-6	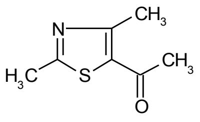	1-(2,4-dimethyl-1,3-thiazol-5-yl)ethan-1-one	0.37 ± 0.1	96.5 ± 10.9	85.8 ± 10.4	62
227	844891-03-8	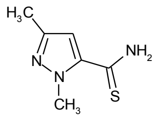	1,3-dimethyl-1H-pyrazole-5-carbothioamide	0.57 ± 0.1	98.4 ± 13.7	106.7 ± 5.8	50.9
228	826-36-8	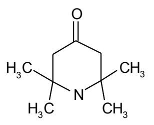	2,2,6,6-tetramethyltetrahydro-4(1H)-pyridinone	-0.07 ± 0.09	98.9 ± 16.8	101.3 ± 5.4	61.9
229	15679-11-5	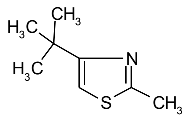	4-(tert-butyl)-2-methyl-1,3-thiazole	0.23 ± 0.07	94.4 ± 6.3	107.5 ± 11.8	65.6
230	609-66-5	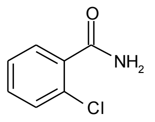	2-chlorobenzamide	-0.22 ± 0.07	96.7 ± 8.2	104.2 ± 8.8	92.9
231	104-11-0	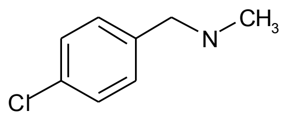	N-(4-chlorobenzyl)-N-methylamine	-0.06 ± 0.07	86.3 ± 3.2	113.3 ± 3.7	106.6

Supplementary Table 1: Screening the Maybridge Rule of Three Fragment Library: TbINO1 differential scanning fluorimetry and cytotoxicity studies against bloodstream form *T.brucei*, HeLa and A549 cells.

233	67952-93-6	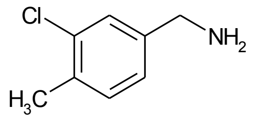	3-chloro-4-methylbenzylamine	0.11 ± 0.08	27.7 ± 4.2	**91.7 ± 12.6**	163.3
234	369-33-5	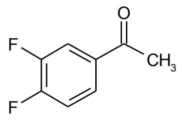	1-(3,4-difluorophenyl)ethan-1-one	0.14 ± 0.08	98.7 ± 6.5	**90.3 ± 14**	73.9
235	553-26-4	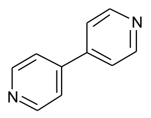	4,4'-dipyridyl	0.2 ± 0.07	95 ± 4.3	63.7 ± 17.4	54.8
237	5928-51-8	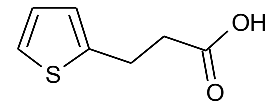	3-(2-thienyl)propanoic acid	0.22 ± 0.07	**85.3 ± 11.8**	113 ± 7.8	70.9
238	58414-52-1	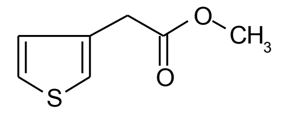	methyl 2-(3-thienyl)acetate	0.39 ± 0.07	**103.4 ± 4**	**111.2 ± 9.9**	64.7
239	4651-97-2	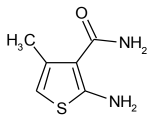	2-amino-4-methylthiophene-3-carboxamide	2.05 ± 0.05	22.9 ± 7	**95.8 ± 9.3**	69.8
240	63155-04-4	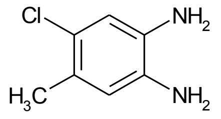	4-chloro-5-methylbenzene-1,2-diamine		28.9 ± 7.8	12.1 ± 8.7	14.7
241	22288-78-4	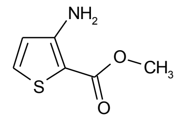	methyl 3-aminothiophene-2-carboxylate	0.38 ± 0.09	**81.2 ± 4.1**	94.5 ± 13	107.8
242	4651-81-4	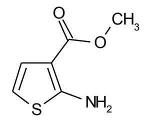	methyl 2-aminothiophene-3-carboxylate	0.34 ± 0.08	**74 ± 4.7**	**37 ± 6.1**	80.5
243	13797-62-1	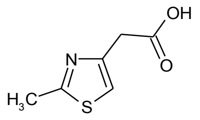	2-(2-methyl-1,3-thiazol-4-yl)acetic acid	-0.23 ± 0.07	97.4 ± 8.3	**99.5 ± 1.1**	113.1
244	69363-85-5	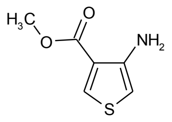	methyl 4-aminothiophene-3-carboxylate	0.61 ± 0.11	39.5 ± 14.5	**78.7 ± 17.9**	90.3
245	1126-09-6	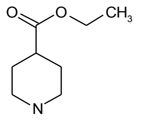	ethyl piperidine-4-carboxylate	-0.04 ± 0.08	93.2 ± 7.2	**97.8 ± 3.2**	99
246	95-03-4	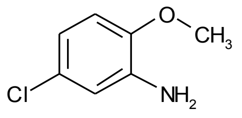	5-chloro-2-methoxyaniline	-0.25 ± 0.06	85.4 ± 17.2	112.6 ± 16.7	113
247	5118-06-9	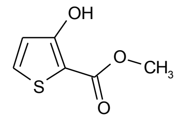	methyl 3-hydroxythiophene-2-carboxylate	0.2 ± 0.07	88.6 ± 3.7	95.4 ± 6.1	108.7
248	80866-95-1	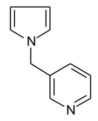	3-(1H-pyrrol-1-ylmethyl)pyridine	0 ± 0.08	72.1 ± 13.2	91.3 ± 11.9	86.6
249	78210-51-2	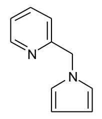	2-(1H-pyrrol-1-ylmethyl)pyridine	-0.36 ± 0.08	**79.2 ± 9.3**	93.1 ± 19.9	83.6
250	52768-17-9	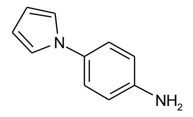	4-(1H-pyrrol-1-yl)aniline	-0.4 ± 0.08	32.4 ± 12.8	107.2 ± 16.6	89.1
251	89353-42-4	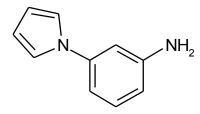	3-(1H-pyrrol-1-yl)aniline	0.15 ± 0.05	29.2 ± 8.7	**80.5 ± 3.4**	79.8
252	174223-29-1	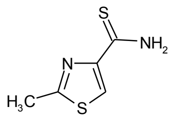	2-methyl-1,3-thiazole-4-carbothioamide	0.7 ± 0.07	92.9 ± 6.6	90.3 ± 10.7	101.7
253	23576-81-0	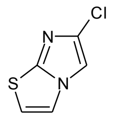	6-chloroimidazo[2,1-b][1,3]thiazole	-0.15 ± 0.08	52.8 ± 5.5	**82.1 ± 0**	85.1

Supplementary Table 1: Screening the Maybridge Rule of Three Fragment Library: TbINO1 differential scanning fluorimetry and cytotoxicity studies against bloodstream form *T.brucei*, HeLa and A549 cells.

255	100516-88-9	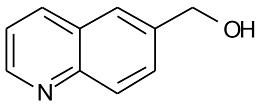	6-quinolinylmethanol	-0.09 ± 0.08	48.1 ± 12	**89.6 ± 6.8**	84
256	1780-17-2	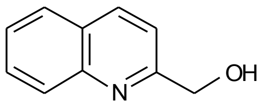	2-quinolinylmethanol	2.2 ± 0.06	21.7 ± 6	99.2 ± 19.5	119.1
257	59147-02-3	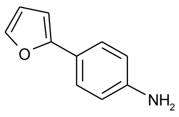	4-(2-furyl)aniline	0.83 ± 0.04	29.3 ± 8.5	**17 ± 12.7**	75.7
258	607-66-9	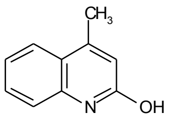	4-methyl-2-quinolinol	0.17 ± 0.06	92.9 ± 6.8	92.7 ± 13.6	90.7
259	143659-19-2	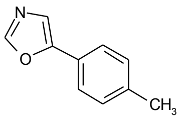	5-(4-methylphenyl)-1,3-oxazole	-0.39 ± 0.08	**90.2 ± 12.5**	88.4 ± 2.5	88.1
260	54705-91-8	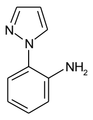	2-(1H-pyrazol-1-yl)aniline	-0.06 ± 0.07	**80.8 ± 0.7**	98.1 ± 5.6	97.3
261	300690-74-8	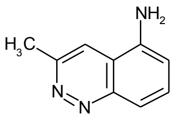	3-methylcinnolin-5-amine	1.83 ± 0.11	21.7 ± 8.5	**62.5 ± 14.7**	74.1
262	26286-54-4	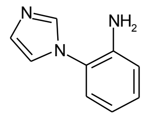	2-(1H-imidazol-1-yl)aniline	0.62 ± 0.08	72.4 ± 20	66.5 ± 16.1	76
264	72235-56-4	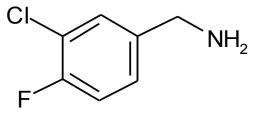	3-chloro-4-fluorobenzylamine	-0.34 ± 0.07	29 ± 5.5	**47.5 ± 13.5**	88.4
265	92-48-8	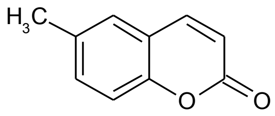	6-methyl-2H-chromen-2-one	-0.11 ± 0.07	91.1 ± 18.7	95.8 ± 17.2	63.8
266	10041-02-8	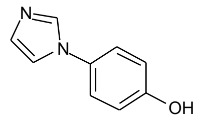	4-(1H-imidazol-1-yl)phenol	-0.1 ± 0.06	**81.3 ± 12.5**	83 ± 19.8	107.9
267	157837-31-5	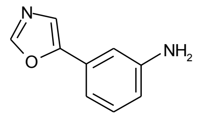	3-(1,3-oxazol-5-yl)aniline	0.13 ± 0.09	**71.1 ± 9.8**	75 ± 15.2	79.8
268	176445-79-7	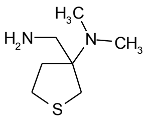	3-(aminomethyl)-N,N-dimethyltetrahydro-3-thiophenamine	0.32 ± 0.07	57.8 ± 19.5	88.4 ± 17.9	87.3
269	139512-70-2	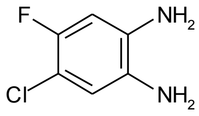	4-chloro-5-fluorobenzene-1,2-diamine	-0.92 ± 0.08	25.1 ± 10.1	23.4 ± 14.8	50.4
270	455-14-1	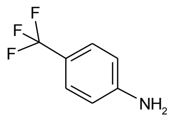	4-(trifluoromethyl)aniline	-4.73 ± 0.19	25.8 ± 6.6	21.8 ± 14.5	103.6
271	1670-82-2	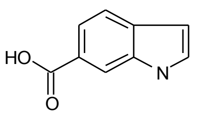	1H-indole-6-carboxylic acid	0.06 ± 0.08	**90.5 ± 2.4**	**110.8 ± 16.2**	75.6
272	1076-59-1	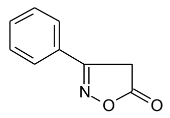	3-phenyl-4,5-dihydroisoxazol-5-one	3.56 ± 0.06	**60.1 ± 21**	**89.4 ± 3.9**	61.5
273	771-50-6	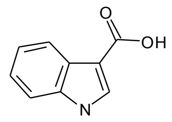	1H-indole-3-carboxylic acid	0.01 ± 0.05	88.6 ± 8.8	**85.4 ± 12.2**	61.8
274	6587-24-2	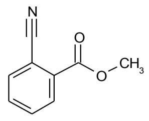	methyl 2-cyanobenzoate	-0.45 ± 0.07	91.2 ± 14.9	91 ± 19.6	64
275	1477-50-5	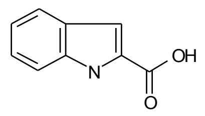	1H-indole-2-carboxylic acid	-0.26 ± 0.08	83 ± 15.6	89.7 ± 16.4	67.3

Supplementary Table 1: Screening the Maybridge Rule of Three Fragment Library: TbINO1 differential scanning fluorimetry and cytotoxicity studies against bloodstream form *T.brucei* , HeLa and A549 cells.

276	81329-31-9	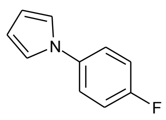	1-(4-fluorophenyl)-1H-pyrrole	0.75 ± 0.08	**45.2 ± 16**	**103.9 ± 16.6**	70.2
277	526-55-6	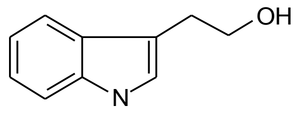	2-(1H-indol-3-yl)ethan-1-ol	1.12 ± 0.06	31.4 ± 8.9	**66.3 ± 1.7**	59.9
278	199590-00-6	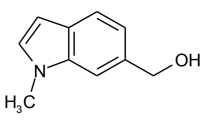	(1-methyl-1H-indol-6-yl)methanol	#VALUE!	28.8 ± 10.8	18.2 ± 10.4	47.8
279	3349-64-2	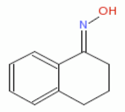	1,2,3,4-tetrahydronaphthalen-1-one oxime	0.76 ± 0.08	18.8 ± 5.8	19.9 ± 19.7	16.9
280	3470-53-9	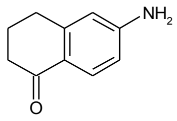	6-amino-1,2,3,4-tetrahydronaphthalen-1-one	0.82 ± 0.09	**42.8 ± 6.8**	40.5 ± 19.2	87.3
281	20028-40-4	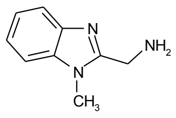	(1-methyl-1H-benzimidazol-2-yl)methylamine	0.7 ± 0.04	27.3 ± 7.7	47.6 ± 19.8	53.8
282	626-43-7	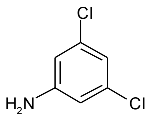	3,5-dichloroaniline	-0.76 ± 0.06	**78.4 ± 11.9**	86.4 ± 8.5	66.5
283	74784-70-6	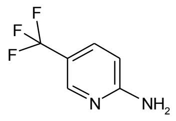	5-(trifluoromethyl)pyridin-2-amine	-0.64 ± 0.08	99.7 ± 14.7	92.7 ± 5.3	63.6
284	75611-06-2	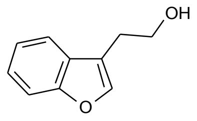	2-benzo[b]furan-3-ylethanol	-0.19 ± 0.07	68.3 ± 14.3	58.7 ± 17.4	63.3
285	58536-48-4	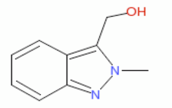	(2-methyl-2H-indazol-3-yl)methanol	0.29 ± 0.07	70.7 ± 13.2	73.4 ± 10.6	67.6
286	1578-96-7	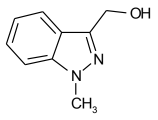	(1-methyl-1H-indazol-3-yl)methanol	-0.31 ± 0.07	**65.3 ± 18**	**90.9 ± 3.1**	73.2
287	30489-44-2	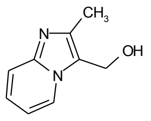	(2-methylimidazo[1,2-a]pyridin-3-yl)methanol	-0.44 ± 0.08	84.7 ± 18.1	76.2 ± 14	79.7
288	2932-65-2	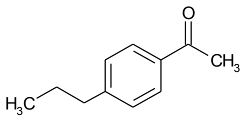	1-(4-propylphenyl)ethan-1-one	0.22 ± 0.08	**33.5 ± 0.5**	72.2 ± 17.1	90.6
289	21627-58-7	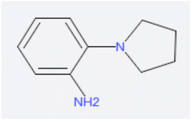	2-pyrrolidin-1-ylaniline		20.1 ± 5.4	11.4 ± 9.5	23.8
290	59337-89-2	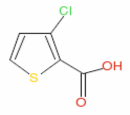	3-chlorothiophene-2-carboxylic acid	-0.21 ± 0.08	**93.3 ± 13.4**	86.9 ± 14.3	117.3
292	78348-24-0	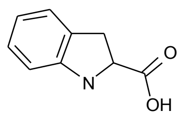	indoline-2-carboxylic acid	1.54 ± 0.11	32.9 ± 9.3	37.6 ± 25	78.3
293	850375-00-7	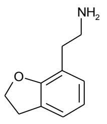	2,3-Dihydrobenzo[b]furan-7-ethylamine	-3.7 ± 0.06	31.2 ± 11.9	**10.8 ± 0.1**	19.8
294	56540-52-4	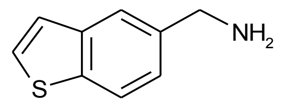	1-benzothiophen-5-ylmethylamine	-0.66 ± 0.07	49.4 ± 20	**98.5 ± 13.1**	70.6
295	177545-13-0	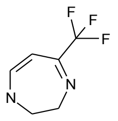	5-(trifluoromethyl)-2,3-dihydro-1H-1,4-diazepine	-0.26 ± 0.07	**94.6 ± 14.4**	75.2 ± 18.7	72.1
296	436-72-6	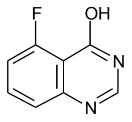	5-fluoroquinazolin-4-ol	-0.28 ± 0.08	80.2 ± 9.5	**92.7 ± 1.9**	63.7

Supplementary Table 1: Screening the Maybridge Rule of Three Fragment Library: TbINO1 differential scanning fluorimetry and cytotoxicity studies against bloodstream form *T.brucei* , HeLa and A549 cells.

297	35700-40-4	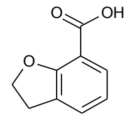	2,3-dihydrobenzo[b]furan-7-carboxylic acid	-0.25 ± 0.08	**101.8 ± 16**	78.8 ± 7.5	74.9
299	83278-86-8	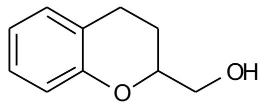	3,4-dihydro-2H-chromen-2-ylmethanol	-0.6 ± 0.08	66.4 ± 16.2	85.1 ± 14.7	80.3
300	76727-28-1	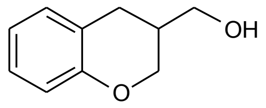	3,4-dihydro-2H-chromen-3-ylmethanol	-0.43 ± 0.08	73.2 ± 12.1	90 ± 16.3	112.9
301	17890-56-1	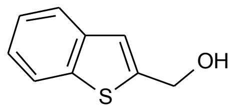	1-benzothiophen-2-ylmethanol	0.01 ± 0.05	68.5 ± 7.6	90.7 ± 4.5	71
302	2941-62-0	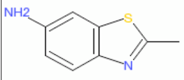	2-methyl-1,3-benzothiazol-6-ylamine	-0.01 ± 0.08	58 ± 4.6	71.9 ± 12.4	62.3
303	6375-17-3	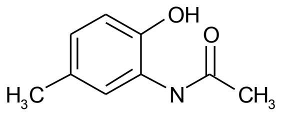	N1-(2-hydroxy-5-methylphenyl)acetamide	0.15 ± 0.07	**82.9 ± 18.1**	95.3 ± 9.8	45
304	18595-14-7	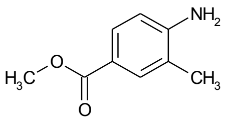	methyl 4-amino-3-methylbenzoate	-0.44 ± 0.08	87 ± 11.8	100.5 ± 8.9	63.4
305	15205-27-3	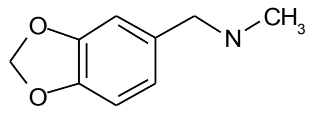	N-(1,3-benzodioxol-5-ylmethyl)-N-methylamine	1.12 ± 0.05	49.3 ± 11.2	56.8 ± 29.4	58.3
306	17413-10-4	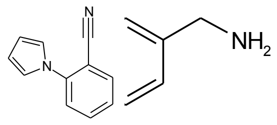	2,3-dihydro-1,4-benzodioxin-6-ylmethylamine	-0.14 ± 0.06	29.2 ± 8	**109.2 ± 5.6**	72.7
307	82756-74-9	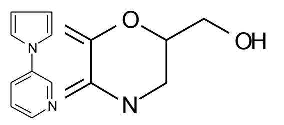	3,4-dihydro-2H-1,4-benzoxazin-2-ylmethanol	0.56 ± 0.06	**76.9 ± 15.1**	81 ± 17.2	70.8
308	175136-34-2	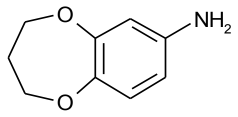	3,4-dihydro-2H-1,5-benzodioxepin-7-amine	-0.38 ± 0.08	**110.4 ± 16.6**	**98.7 ± 6.1**	82.8
309	4442-59-5	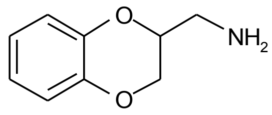	2,3-dihydro-1,4-benzodioxin-2-ylmethylamine	-0.06 ± 0.07	24.7 ± 6.9	12.3 ± 8.8	68.9
310	13673-63-7	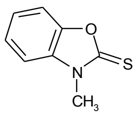	3-methyl-1,3-benzoxazole-2(3H)-thione	-0.38 ± 0.07	91.4 ± 3.7	102.6 ± 14.3	104.3
311	131337-81-0	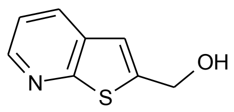	thieno[2,3-b]pyridin-2-ylmethanol	0.8 ± 0.08	70.8 ± 9	92 ± 11.8	77.5
312	37859-42-0	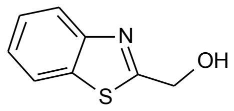	1,3-benzothiazol-2-ylmethanol	-0.44 ± 0.09	73.2 ± 12.2	80.7 ± 16.2	64.4
313	96799-03-0	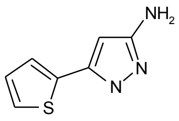	5-thien-2-yl-1H-pyrazol-3-amine	0.69 ± 0.08	52.4 ± 7	100.8 ± 18.9	81.7
314	65865-28-3	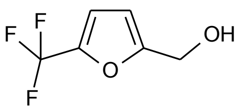	[5-(trifluoromethyl)-2-furyl]methanol	-0.28 ± 0.08	99.1 ± 0.6	113.8 ± 8.2	76.9
315	1481-02-3	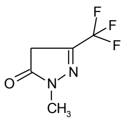	1-methyl-3-(trifluoromethyl)-4,5-dihydro-1H-pyrazol-5-one	0.09 ± 0.08	103.3 ± 15.9	**109.2 ± 4.4**	88.3
316	119022-51-4	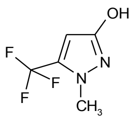	1-methyl-5-(trifluoromethyl)-1H-pyrazol-3-ol	-0.59 ± 0.08	91.7 ± 8.2	111.8 ± 17.2	95.3
317	75091-99-5	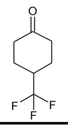	4-(trifluoromethyl)cyclohexan-1-one	-0.33 ± 0.08	106 ± 12.4	110.7 ± 7.2	90.5

Supplementary Table 1: Screening the Maybridge Rule of Three Fragment Library: TbINO1 differential scanning fluorimetry and cytotoxicity studies against bloodstream form *T.brucei* , HeLa and A549 cells.

318	66892-34-0	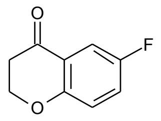	6-fluorochroman-4-one	-0.19 ± 0.07	88.6 ± 12.2	104.4 ± 16.4	99
319	3663-82-9	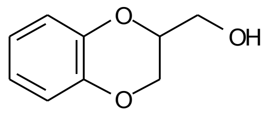	2,3-dihydro-1,4-benzodioxin-2-ylmethanol	-0.54 ± 0.08	77.2 ± 5.4	102.6 ± 5.1	86.4
320	39270-39-8	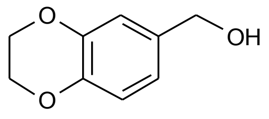	2,3-dihydro-1,4-benzodioxin-6-ylmethanol	-0.73 ± 0.08	83.1 ± 11.5	111.9 ± 16.4	78.8
321	6006-82-2	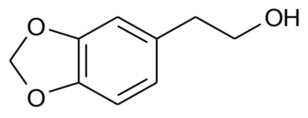	2-(1,3-benzodioxol-5-yl)ethanol	-0.17 ± 0.08	35.2 ± 14.9	**109.4 ± 5.9**	138.3
322	274910-19-9	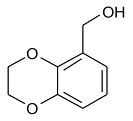	2,3-dihydro-1,4-benzodioxin-5-ylmethanol	-0.16 ± 0.07	90.8 ± 4.4	100.4 ± 14.9	96.4
323	495-78-3	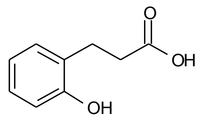	3-(2-hydroxyphenyl)propanoic acid	1.23 ± 0.07	111.4 ± 8.6	113.1 ± 2.8	109.5
324	21170-10-5	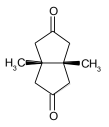	3a,6a-dimethylperhydropentalene-2,5-dione	-0.05 ± 0.06	88.2 ± 0.7	103.2 ± 21.9	94.2
325	4104-75-0	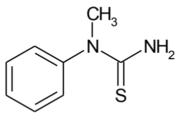	N-methyl-N-phenylthiourea	-0.82 ± 0.06	24 ± 5.4	33.9 ± 11.6	82.7
326	614-78-8	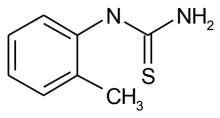	N-(2-methylphenyl)thiourea	-0.03 ± 0.07	**90.2 ± 6.3**	111.4 ± 8.5	124.7
327	859851-02-8	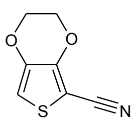	2,3-dihydrothieno[3,4-b][1,4]dioxine-5-carbonitrile	0.12 ± 0.06	89.7 ± 4.3	115.5 ± 14.4	121.1
328	39793-31-2	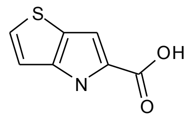	4H-thieno[3,2-b]pyrrole-5-carboxylic acid	2.29 ± 0.1	**51.6 ± 13.3**	105.9 ± 6.9	136.2
329	3275-95-4	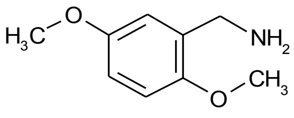	2,5-dimethoxybenzylamine	0.97 ± 0.05	24.1 ± 3.4	**26 ± 9**	155.7
330	2199-44-2	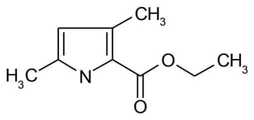	ethyl 3,5-dimethyl-1H-pyrrole-2-carboxylate	0.61 ± 0.06	90.7 ± 1.7	122.4 ± 12.3	155.7
331	121933-59-3	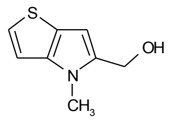	(4-methyl-4H-thieno[3,2-b]pyrrol-5-yl)methanol	1.54 ± 0.07	31 ± 7.1	**93.6 ± 14.9**	120.9
332	13559-66-5	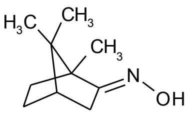	1,7,7-trimethylbicyclo[2.2.1]heptan-2-one oxime	-0.76 ± 0.07	106.5 ± 18.9	110.6 ± 8.3	93.9
333	30129-18-1	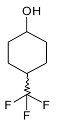	4-(trifluoromethyl)cyclohexan-1-ol	0.47 ± 0.04	99.1 ± 5.6	109.7 ± 4.4	113.6
334	76523-24-5	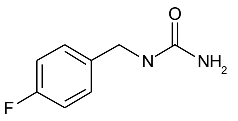	N-(4-fluorobenzyl)urea	-0.33 ± 0.07	92.1 ± 14	**112.4 ± 4.7**	91.8
335	348-40-3	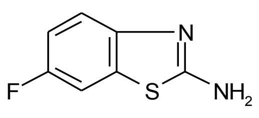	6-fluoro-1,3-benzothiazol-2-amine	-4.93 ± 2.59	37.7 ± 12	70.6 ± 6.1	113.2
336	33265-71-3	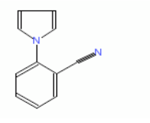	2-(1H-pyrrol-1-yl)benzonitrile		33.1 ± 13	68.5 ± 16.3	134.2
338	112598-77-3	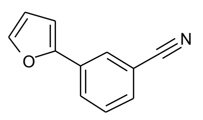	3-(2-furyl)benzonitrile		35.6 ± 7.3	18.5 ± 15.2	57.8

Supplementary Table 1: Screening the Maybridge Rule of Three Fragment Library: TbINO1 differential scanning fluorimetry and cytotoxicity studies against bloodstream form *T.brucei* , HeLa and A549 cells.

339	25699-85-8	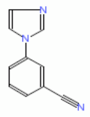	3-(1H-imidazol-1-yl)benzonitrile	0.39 ± 0.06	72.1 ± 7.8	89.8 ± 5.8	134.7
340	25775-03-5	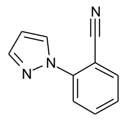	2-(1H-pyrazol-1-yl)benzonitrile	0.18 ± 0.06	78.8 ± 10.1	104.1 ± 16.5	134
342	19932-84-4	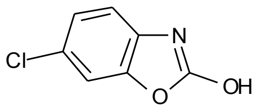	6-chloro-1,3-benzoxazol-2-ol	-0.61 ± 0.06	**111.3 ± 3.1**	94.3 ± 6.5	73.9
343	14099-81-1	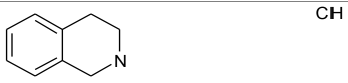	1,2,3,4-tetrahydroisoquinoline hydrochloride	0.37 ± 0.06	**67 ± 10.6**	83.5 ± 7.8	86.3
344	619334-28-0	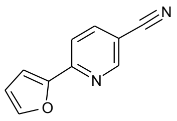	6-(2-furyl)nicotinonitrile	-0.23 ± 0.17	40.4 ± 13.9	48.9 ± 5	103.6
345	257932-29-9	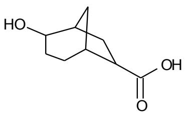	2-hydroxybicyclo[3.2.1]octane-6-carboxylic acid	-0.22 ± 0.07	81.9 ± 2.6	87.4 ± 13	127
346	57976-57-5	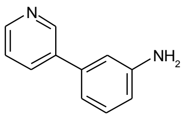	3-pyridin-3-ylaniline		35.6 ± 9.3	73.9 ± 6.1	98.5
347	82261-42-5	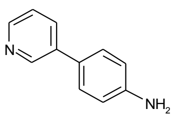	4-pyridin-3-ylaniline	-0.88 ± 0.08	26.4 ± 5.2	79.3 ± 2.8	113.3
348	177202-83-4	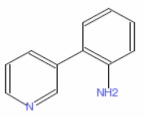	2-pyridin-3-ylaniline	-0.23 ± 0.06	22.2 ± 5.6	18.2 ± 10	85.3
349	690632-11-2	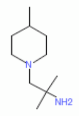	2-methyl-1-(4-methylpiperidino)-2-propanamine	0.53 ± 0.06	103.1 ± 7.3	108.4 ± 7.2	144.1
350	7499-07-2	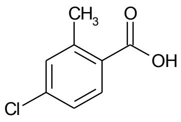	4-chloro-2-methylbenzoic acid	1.05 ± 0.07	**84.2 ± 12.3**	108.5 ± 9.9	159.9
351	5162-82-3	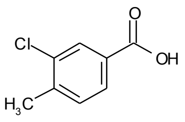	3-chloro-4-methylbenzoic acid	-0.41 ± 0.07	38 ± 12.2	88.2 ± 17.9	112.1
352	610-96-8	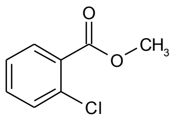	methyl 2-chlorobenzoate	-0.51 ± 0.41	93.2 ± 9.2	95.8 ± 19	76.4
353	69491-59-4	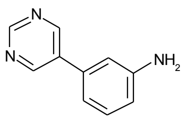	3-pyrimidin-5-ylaniline	0.44 ± 0.06	**69.2 ± 4.2**	78.7 ± 10	56.4
354	85006-31-1	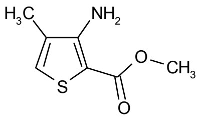	methyl 3-amino-4-methylthiophene-2-carboxylate	-0.64 ± 0.07	81.3 ± 9.1	72.8 ± 8.6	32.3
355	88-19-7	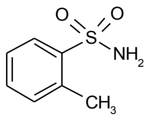	2-methylbenzene-1-sulfonamide	-0.76 ± 0.06	98.8 ± 16	89.1 ± 16.6	87.9
356	6436-59-5	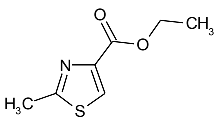	ethyl 2-methyl-1,3-thiazole-4-carboxylate	0.28 ± 0.08	101.4 ± 3.7	106.3 ± 5.3	79.8
357	71705-07-2	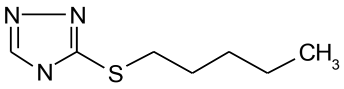	3-(pentylthio)-4H-1,2,4-triazole	-1.1 ± 0.07	23.8 ± 4.9	64.5 ± 8.6	45.3
358	13600-43-6	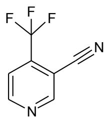	4-(trifluoromethyl)nicotinonitrile	-0.04 ± 0.08	111.9 ± 7.2	103.3 ± 1.7	129.4
359	105184-38-1	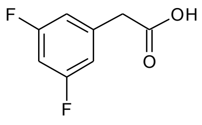	2-(3,5-difluorophenyl)acetic acid	0.13 ± 0.06	95.2 ± 10.8	107.5 ± 13.6	128.3

Supplementary Table 1: Screening the Maybridge Rule of Three Fragment Library: TbINO1 differential scanning fluorimetry and cytotoxicity studies against bloodstream form *T.brucei* , HeLa and A549 cells.

360	140675-42-9	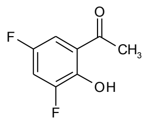	1-(3,5-difluoro-2-hydroxyphenyl)ethan-1-one	1.29 ± 0.05	**98.4 ± 8.8**	103.5 ± 10	149.7
361	859851-01-7	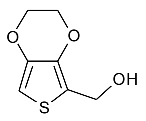	2,3-dihydrothieno[3,4-b][1,4]dioxin-5-ylmethanol	-0.13 ± 0.08	**86.3 ± 8.1**	68.5 ± 12.9	93.7
362	175696-73-8	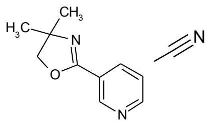	3-pyrrolidin-1-ylbenzonitrile	0.05 ± 0.07	**88.1 ± 5.6**	97.4 ± 19.9	63.3
363	39243-88-4	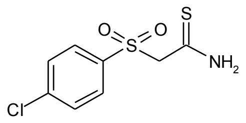	[2-(1H-pyrrol-1-yl)phenyl]methylamine	-0.09 ± 0.06	28.8 ± 7.9	42.6 ± 11.7	45.6
364	368869-95-8	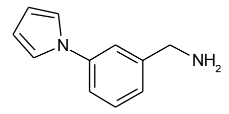	3-(1H-pyrrol-1-yl)benzylamine	-0.1 ± 0.07	23.2 ± 4.2	29.5 ± 12.9	56.9
365	465514-27-6	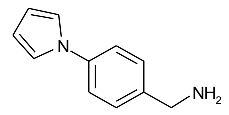	4-(1H-pyrrol-1-yl)benzylamine	-0.51 ± 0.09	22.3 ± 5.1	**50.9 ± 11**	94.4
366	179873-36-0	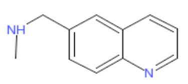	N-methyl-N-(quinolin-6-ylmethyl)amine	-0.12 ± 0.06	41.5 ± 2.8	64.6 ± 11.4	74.6
367	2923-66-2	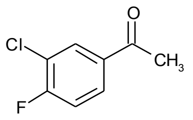	1-(3-chloro-4-fluorophenyl)ethan-1-one	-0.15 ± 0.07	101 ± 10.3	98.7 ± 6.5	58.8
368	486-74-8	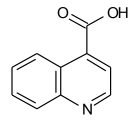	quinoline-4-carboxylic acid	0.33 ± 0.08	96.3 ± 9.9	90.7 ± 7.5	90.3
369	93-10-7	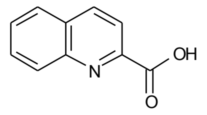	2-quinolinecarboxylic acid	0.1 ± 0.67	**69.2 ± 10.7**	81.3 ± 14.1	152.1
370	10349-57-2	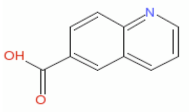	quinoline-6-carboxylic acid	0.57 ± 0.04	**107.3 ± 8.1**	109.3 ± 13.1	151.8
371	61034-86-4	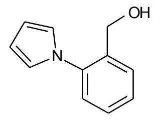	[2-(1H-pyrrol-1-yl)phenyl]methanol	0.36 ± 0.07	38.3 ± 1.4	**88.5 ± 6.4**	87.3
372	143426-51-1	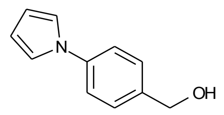	[4-(1H-pyrrol-1-yl)phenyl]methanol	3.12 ± 0.04	**37 ± 14**	**94.9 ± 19.4**	52.9
373	771573-27-4		[4-(2-furyl)phenyl]methylamine	0.48 ± 0.05	**50.5 ± 24.8**	**82.5 ± 14.3**	63.4
374	108166-02-5	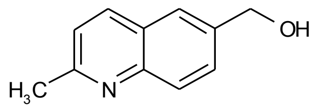	(2-methyl-6-quinolinyl)methanol	0.1 ± 0.06	74.9 ± 15.6	53.2 ± 2.6	46.3
375	56643-85-7	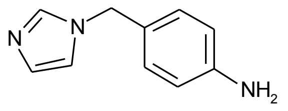	4-(1H-imidazol-1-ylmethyl)aniline	-0.24 ± 0.08	96.3 ± 7.6	88 ± 18.8	90.7
376	10199-50-5	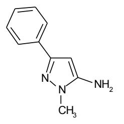	1-methyl-3-phenyl-1H-pyrazol-5-amine	-0.59 ± 0.06	62.2 ± 5.1	82.6 ± 4.6	64.7
377	120107-85-9	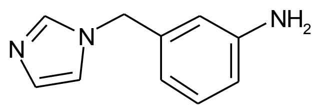	3-(1H-imidazol-1-ylmethyl)aniline	0.19 ± 0.07	93.8 ± 15.8	102.6 ± 12.6	62.4
378	368870-03-5	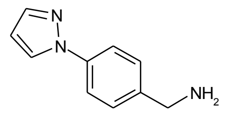	4-(1H-pyrazol-1-yl)benzylamine	0.19 ± 0.06	**46.3 ± 17.2**	100.7 ± 8.3	95.9
379	10442-03-2	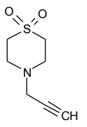	4-(Prop-2-ynyl)thiomorpholine 1,1-dioxide	0.32 ± 0.08	102.9 ± 14.9	95.6 ± 12.9	154

Supplementary Table 1: Screening the Maybridge Rule of Three Fragment Library: TbINO1 differential scanning fluorimetry and cytotoxicity studies against bloodstream form *T.brucei* , HeLa and A549 cells.

380	701-43-9	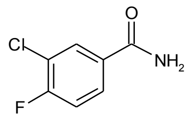	3-chloro-4-fluorobenzamide	0.31 ± 0.06	90.8 ± 9.6	92.2 ± 13.4	95.9
381	6419-36-9	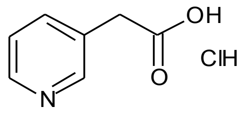	pyridin-3-ylacetic acid hydrochloride	1.08 ± 0.05	**111.3 ± 15.5**	99.2 ± 9.9	105.4
382	16423-54-4	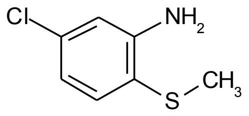	5-chloro-2-(methylthio)aniline	-0.75 ± 0.07	105 ± 13.7	109.8 ± 13.3	60.7
383	17920-85-3	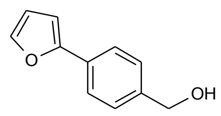	[4-(2-furyl)phenyl]methanol	3.45 ± 0.09	33 ± 7.2	20 ± 10.6	89.6
384	21169-65-3	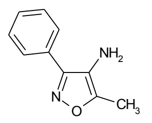	5-methyl-3-phenyl-4-isoxazolamine	-0.09 ± 0.07	**106.8 ± 7.1**	80.8 ± 13.4	75.5
385	143426-49-7	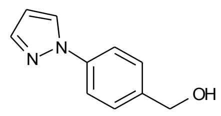	[4-(1H-pyrazol-1-yl)phenyl]methanol	-0.08 ± 0.07	78.3 ± 5	83.4 ± 12.4	117.5
386	86718-08-3	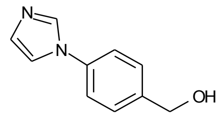	[4-(1H-imidazol-1-yl)phenyl]methanol	-0.75 ± 0.08	**87.6 ± 0.2**	102.8 ± 15.3	101.2
387	56278-50-3	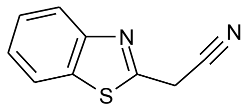	2-(1,3-benzothiazol-2-yl)acetonitrile	1.29 ± 0.14	36 ± 8.3	86.4 ± 10.4	81
388	79887-15-3	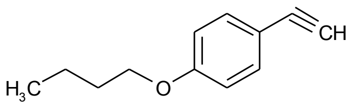	1-butoxy-4-eth-1-ynylbenzene	-0.05 ± 0.07	100.2 ± 10.8	107.3 ± 8	114.5
389	20873-58-9	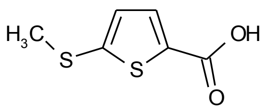	5-(methylthio)thiophene-2-carboxylic acid	0.43 ± 0.05	**85.6 ± 16.6**	104.2 ± 16.4	125.1
390	3300-51-4	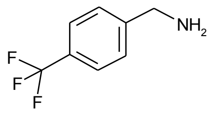	4-(trifluoromethyl)benzylamine	0.38 ± 0.08	24 ± 4.6	**84.4 ± 6.8**	135.7
391	87-51-4	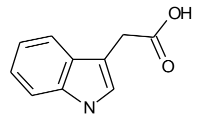	2-(1H-indol-3-yl)acetic acid	1.67 ± 0.08	38.8 ± 10.1	**42.6 ± 6.4**	133.7
392	942-24-5	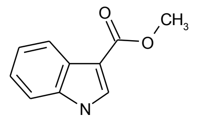	methyl 1H-indole-3-carboxylate	0.22 ± 0.06	90.6 ± 14.9	109.9 ± 3.7	88.1
393	1619-37-0	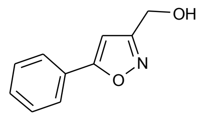	(5-phenylisoxazol-3-yl)methanol	-0.58 ± 0.07	62.5 ± 6.9	64.8 ± 13.5	60
394	186129-25-9	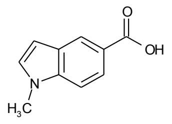	1-methyl-1H-indole-5-carboxylic acid	0 ± 0.08	**111.3 ± 0.3**	95 ± 12.2	82
395	1677-46-9	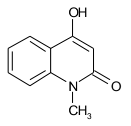	4-hydroxy-1-methyl-1,2-dihydroquinolin-2-one	1.62 ± 0.03	54.3 ± 16.4	**93 ± 0.3**	174.4
396	93247-78-0	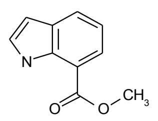	methyl 1H-indole-7-carboxylate	0.12 ± 0.11	35.3 ± 9.5	36.5 ± 11.9	164.1
397	352018-88-3	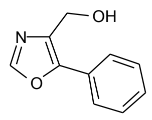	(5-phenyl-1,3-oxazol-4-yl)methanol	-0.06 ± 0.08	24.2 ± 6.1	18.2 ± 7.9	56.4
398	202745-73-1	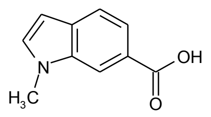	1-methyl-1H-indole-6-carboxylic acid	-0.02 ± 0.08	**88.8 ± 14.9**	109.2 ± 10.3	101.4
399	90924-06-4	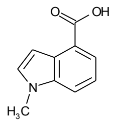	1-methyl-1H-indole-4-carboxylic acid	0.63 ± 0.07	77.4 ± 9.9	99.3 ± 14.2	123.2

Supplementary Table 1: Screening the Maybridge Rule of Three Fragment Library: TbINO1 differential scanning fluorimetry and cytotoxicity studies against bloodstream form *T.brucei* , HeLa and A549 cells.

400	59856-06-3	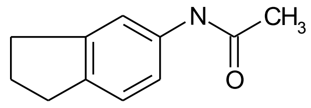	N1-(2,3-dihydro-1H-inden-5-yl)acetamide	0.12 ± 0.07	**81.6 ± 17.2**	23.9 ± 13.6	99.3
401	124491-38-9	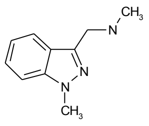	N-methyl-N-[(1-methyl-1H-indazol-3-yl)methyl]amine	-0.3 ± 0.07	**59.8 ± 8.8**	92.5 ± 7.4	91.7
402	58729-31-0	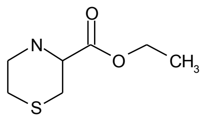	ethyl thiomorpholine-3-carboxylate	0.45 ± 0.08	**113.7 ± 12.9**	107.3 ± 18.3	95.2
403	92057-12-0	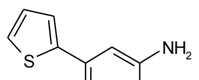	3-(2-thienyl)aniline	0.04 ± 0.04	32 ± 5.9	49.1 ± 15.7	52.3
404	161886-96-0	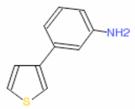	3-thien-3-ylaniline	0.17 ± 0.05	39.3 ± 5.7	89.4 ± 17.7	83.3
405	39989-43-0	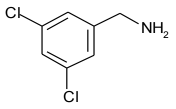	3,5-dichlorobenzylamine	-0.53 ± 0.08	30.8 ± 5.6	**77.7 ± 13.9**	84.1
407	346-06-5	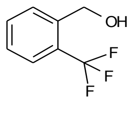	[2-(trifluoromethyl)phenyl]methanol	-0.43 ± 0.08	101.4 ± 9.7	103.7 ± 13.9	90.2
409	29427-69-8	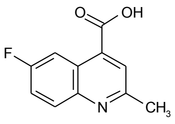	3-oxoindane-1-carboxylic acid	0.03 ± 0.07	118.6 ± 18.3	106.7 ± 10.9	92
410	64175-51-5	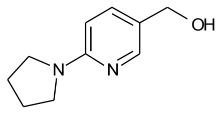	benzo[b]furan-3-ylacetic acid	0.8 ± 0.07	55.1 ± 19.3	54.1 ± 10.3	97.4
413	2359-60-6	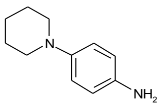	4-piperidinoaniline	#VALUE!	26 ± 4.8	11.6 ± 5.1	53.2
414	114365-04-7	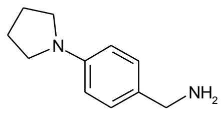	[4-(1-pyrrolidinyl)phenyl]methanamine	0.14 ± 0.07	31.2 ± 6.3	68 ± 13.7	96.8
415	183365-31-3	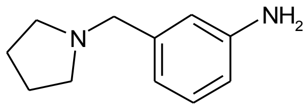	3-(pyrrolidin-1-ylmethyl)aniline	0.34 ± 0.06	47.4 ± 18.4	**57.8 ± 4.5**	107.4
416	76883-37-9	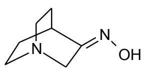	quinuclidin-3-one oxime hydrochloride	-0.24 ± 0.08	109.1 ± 15	107.2 ± 9.6	109.3
418	1535-75-7	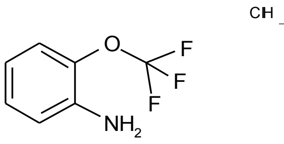	2-(trifluoromethoxy)aniline	0.14 ± 0.07	**86.8 ± 2.2**	**99.1 ± 14.1**	97.9
419	1535-73-5	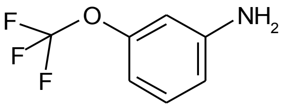	3-(trifluoromethoxy)aniline	-0.13 ± 0.08	104.2 ± 2.1	104.9 ± 11.1	85
420	251649-52-2	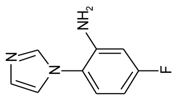	5-fluoro-2-(1H-imidazol-1-yl)aniline	0.36 ± 0.07	77.6 ± 18.8	98.2 ± 10.9	100.1
421	90719-32-7	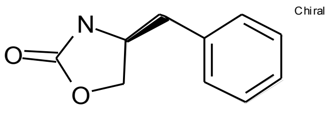	(S)-4-BENZYL-2-OXAZOLIDINONE	-0.54 ± 0.08	79.1 ± 9.8	86 ± 5.1	80.2
422	96056-64-3	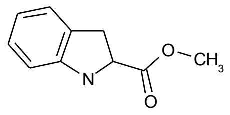	methyl 2-indolinecarboxylate	-0.42 ± 0.1	32.3 ± 8.4	11.1 ± 5.7	40.1
423	17467-15-1	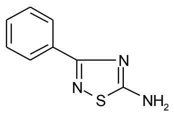	3-phenyl-1,2,4-thiadiazol-5-amine	-0.1 ± 0.08	59.6 ± 12.3	82.7 ± 3.9	52.7
424	27182-54-3	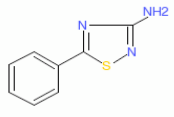	5-phenyl-1,2,4-thiadiazol-3-amine	-1.96 ± 0.19	**106.8 ± 4.7**	62.2 ± 15.4	60.1

Supplementary Table 1: Screening the Maybridge Rule of Three Fragment Library: TbINO1 differential scanning fluorimetry and cytotoxicity studies against bloodstream form *T.brucei* , HeLa and A549 cells.

426	20330-99-8	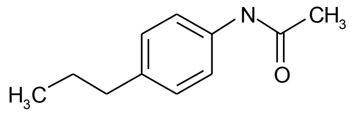	N1-(4-propylphenyl)acetamide	-2.28 ± 0.07	**104.3 ± 11.3**	89.8 ± 10.8	71.7
427	676245-12-8	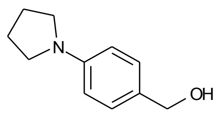	[4-(1-pyrrolidinyl)phenyl]methanol	0.35 ± 0.06	27.3 ± 5.7	59.8 ± 14.4	64.2
428	859850-72-9	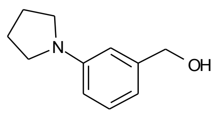	(3-pyrrolidin-1-ylphenyl)methanol	0.44 ± 0.07	37.2 ± 16.5	**12.6 ± 5.6**	81.2
429	13339-02-1	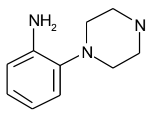	2-piperazin-1-ylaniline	-0.13 ± 0.07	40.2 ± 13.4	**78.5 ± 7.7**	54.3
430	2879-20-1	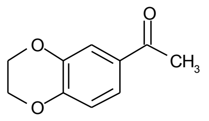	1-(2,3-dihydro-1,4-benzodioxin-6-yl)ethan-1-one	0.13 ± 0.08	**90.9 ± 7.7**	91.1 ± 12.8	101.8
431	115822-57-6	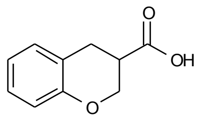	3-chromanecarboxylic acid	-0.1 ± 0.07	95.2 ± 14.3	102.3 ± 5.9	95.8
432	5381-25-9	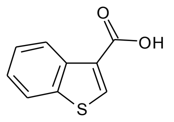	1-benzothiophene-3-carboxylic acid	-0.41 ± 0.08	83.1 ± 17.9	88.5 ± 12.2	55.1
434	38002-89-0	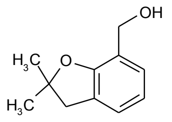	(2,2-dimethyl-2,3-dihydro-1-benzofuran-7-yl)methanol	-0.23 ± 0.06	30 ± 6.2	80.2 ± 12	51.1
435	2524-67-6	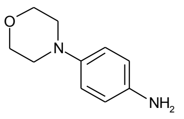	4-morpholinoaniline	-6.78 ± 0.07	21.3 ± 3.7	10.2 ± 6.9	33.5
436	5585-33-1	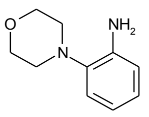	2-morpholinoaniline	-0.36 ± 0.07	**83 ± 6.1**	79.1 ± 5.8	56.4
437	690632-01-0	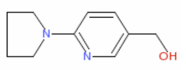	[6-(1-pyrrolidinyl)-3-pyridinyl]methanol	-0.2 ± 0.08	29.4 ± 4.1	9.3 ± 4.8	44.3
438	906352-65-6	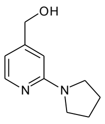	(2-pyrrolidin-1-ylpyrid-4-yl)methanol	-0.24 ± 0.08	**73.4 ± 14**	102.6 ± 14.9	71.9
439	70298-89-4	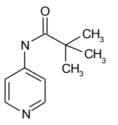	2,2-dimethyl-N-(4-pyridinyl)propanamide	-0.24 ± 0.07	61 ± 18.6	54.6 ± 4.5	65
440	690632-85-0	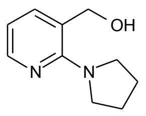	[2-(1-pyrrolidinyl)-3-pyridinyl]methanol	0.78 ± 0.08	44.3 ± 11.8	**74.6 ± 7.5**	94
441	159724-40-0	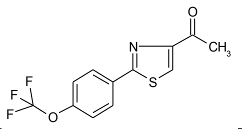	3-morpholin-4-ylaniline	0.27 ± 0.08	52.6 ± 19.5	77.7 ± 16.7	85
442	22962-49-8	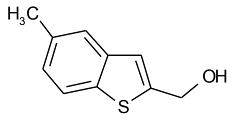	5-Methylbenzo[b]thiophene-2-methanol	-0.17 ± 0.07	**30.5 ± 6**	71.3 ± 3.5	53.7
443	25877-30-9	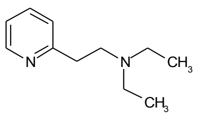	N,N-diethyl-N-[2-(2-pyridyl)ethyl]amine	-0.31 ± 0.07	**98.7 ± 8.7**	90.9 ± 9.8	49.2
444	3622-35-3	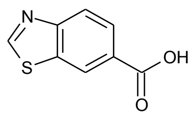	1,3-benzothiazole-6-carboxylic acid	0.73 ± 0.06	111.5 ± 19.8	92 ± 9.7	63.5
445	59944-76-2	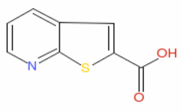	thieno[2,3-b]pyridine-2-carboxylic acid	0.61 ± 0.08	97 ± 18.5	88 ± 8.1	45.2
446	5438-70-0	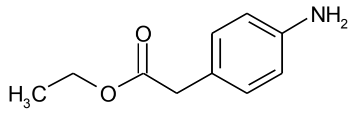	ethyl 2-(4-aminophenyl)acetate	-0.6 ± 0.07	**110.8 ± 15.4**	80.1 ± 14.8	63.2

Supplementary Table 1: Screening the Maybridge Rule of Three Fragment Library: TbINO1 differential scanning fluorimetry and cytotoxicity studies against bloodstream form *T.brucei* , HeLa and A549 cells.

447	27292-49-5	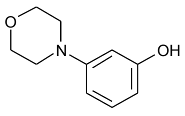	3-morpholinophenol	-0.1 ± 0.08	**70.4 ± 22.1**	77.8 ± 11.8	62.8
448	41536-44-1	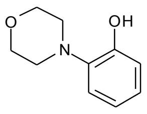	2-morpholinophenol	-0.04 ± 0.08	101 ± 18.8	93.3 ± 6.7	71.1
450	118430-78-7	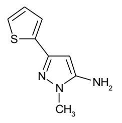	1-methyl-3-(2-thienyl)-1H-pyrazol-5-amine	1.1 ± 0.06	55.3 ± 16.7	89.3 ± 5.5	90.7
451	18927-05-4	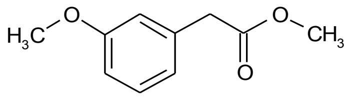	methyl 2-(3-methoxyphenyl)acetate	-0.68 ± 0.08	**50.7 ± 17.1**	100.7 ± 9.6	93.2
452	6342-77-4	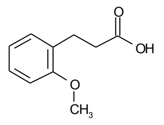	3-(2-methoxyphenyl)propanoic acid	-0.61 ± 0.08	49.2 ± 15	97.3 ± 7.2	61.6
453	62823-14-7	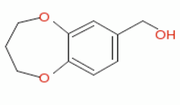	3,4-dihydro-2H-1,5-benzodioxepin-7-ylmethanol	-0.59 ± 0.08	**111.4 ± 1.5**	86.6 ± 3.2	51.6
454	5766-40-5	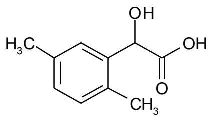	2-(2,5-dimethylphenyl)-2-hydroxyacetic acid	0.02 ± 0.06	**98.7 ± 11.9**	96.8 ± 12.7	60.8
456	39065-54-8	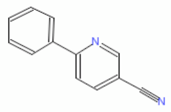	6-phenylnicotinonitrile	-1.66 ± 0.17	31.1 ± 4.1	33.9 ± 6.9	67.1
457	2252-63-3	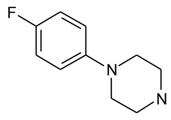	1-(4-fluorophenyl)piperazine	-0.57 ± 0.08	32.3 ± 5.6	12.7 ± 10.1	74.7
458	868755-65-1	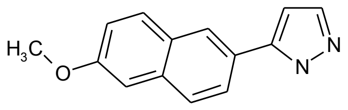	[5-(2-furyl)thien-2-yl]methanol	0.6 ± 0.02	71.4 ± 18	94 ± 12.5	73.6
459	852228-02-5	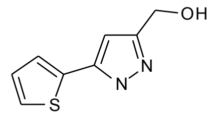	(5-thien-2-yl-1H-pyrazol-3-yl)methanol		**41.2 ± 2.9**	**27.8 ± 10.9**	85
460	1200-13-1	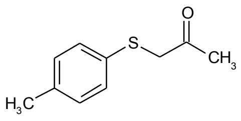	1-[(4-methylphenyl)thio]acetone	0.46 ± 0.07	34.1 ± 5.3	97 ± 2.5	96.9
461	541-69-5	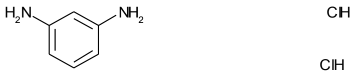	benzene-1,3-diamine dihydrochloride	-0.49 ± 0.08	97.3 ± 16.9	105.6 ± 10.7	91.8
462	194491-44-6	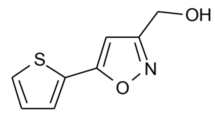	[5-(2-thienyl)-3-isoxazolyl]methanol	-0.72 ± 0.08	**80.7 ± 24**	75.6 ± 5.9	76.2
463	118465-49-9	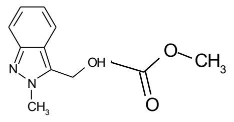	methyl 6H-thieno[2,3-b]pyrrole-5-carboxylate	-0.89 ± 0.09	**85.3 ± 19.7**	101.3 ± 11	59.7
464	841222-62-6	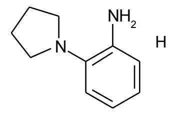	4-methyl-4H-thieno[3,2-b]pyrrole-5-carboxylic acid		**82 ± 5.9**	93.5 ± 4.2	89.4
465	103-46-8	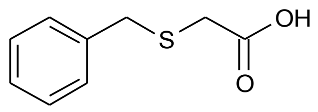	2-(benzylthio)acetic acid	-0.33 ± 0.08	**110.7 ± 12.3**	99 ± 9.3	67.9
466	17277-58-6	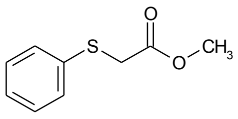	methyl 2-(phenylthio)acetate	-0.65 ± 0.08	93.8 ± 5.2	101.8 ± 4.8	84.7
467	423769-75-9	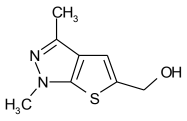	(1,3-dimethyl-1H-thieno[2,3-c]pyrazol-5-yl)methanol	0.35 ± 0.09	65.6 ± 13.2	67.5 ± 16.4	70.7
468	35090-94-9	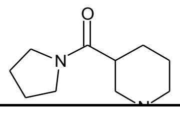	3-piperidinyl(1-pyrrolidinyl)methanone	-0.03 ± 0.08	93.4 ± 18.6	**109.8 ± 13.3**	80.7

Supplementary Table 1: Screening the Maybridge Rule of Three Fragment Library: TbINO1 differential scanning fluorimetry and cytotoxicity studies against bloodstream form *T.brucei* , HeLa and A549 cells.

469	35090-95-0	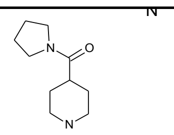	4-piperidinyl(1-pyrrolidinyl)methanone	-0.35 ± 0.07	99.9 ± 7.5	99.5 ± 8.7	86.1
471	106981-51-5	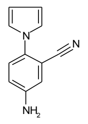	5-amino-2-(1H-pyrrol-1-yl)benzonitrile	0.1 ± 0.08	42.4 ± 15.4	89.3 ± 18.8	81.4
472	59865-82-6	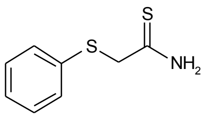	2-(phenylthio)ethanethioamide	-3.57 ± 0.04	43 ± 11	76.5 ± 11.2	86
473	5202-86-8	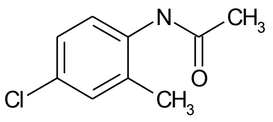	4-chloro-2-methylacetanilide	-0.4 ± 0.07	**86 ± 1**	108.7 ± 11.9	76.1
474	33305-08-7	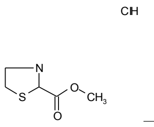	methyl 1,3-thiazolane-2-carboxylate hydrochloride	2.81 ± 0.1	86.7 ± 16.4	108.2 ± 15.9	87
475	56-92-8	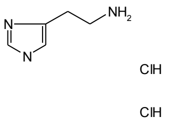	2-(1H-imidazol-4-yl)ethylamine dihydrochloride	-0.03 ± 0.08	**118.7 ± 4.4**	95.9 ± 6.6	80.3
476	306934-89-4	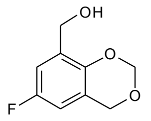	(6-fluoro-4H-1,3-benzodioxin-8-yl)methanol	0.01 ± 0.07	33.3 ± 4.3	102.6 ± 9.4	78.9
477	14742-32-6	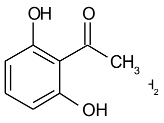	2-amino-5-phenyl-3-furonitrile	0.04 ± 0.1	**93.4 ± 0.3**	98.3 ± 18.6	97.4
478	4653-08-1	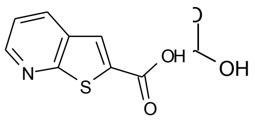	4-oxo-4-(2-thienyl)butanoic acid	2.58 ± 0.05	**103.8 ± 13.1**	105.8 ± 9.3	92.3
479	1723-27-9	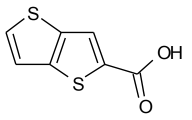	thieno[3,2-b]thiophene-2-carboxylic acid	0.21 ± 0.07	**91.9 ± 9**	108.9 ± 11.4	102.5
481	33617-67-3	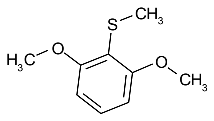	1,3-dimethoxy-2-(methylthio)benzene	0.07 ± 0.08	**82.6 ± 6.8**	104.2 ± 17.6	102.7
482	108372-48-1	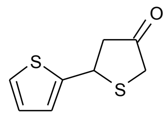	5-(2-thienyl)tetrahydrothiophen-3-one	-1.99 ± 0.06	40.9 ± 5.9	**115.5 ± 6.7**	111.9
484	56525-63-4	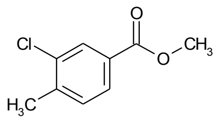	methyl 3-chloro-4-methylbenzoate	0.1 ± 0.07	98.3 ± 14	112.3 ± 12	119.6
486	43088-67-1	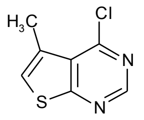	4-chloro-5-methylthieno[2,3-d]pyrimidine	0.24 ± 0.07	125.4 ± 5	100.7 ± 12.2	110.6
487	33143-29-2	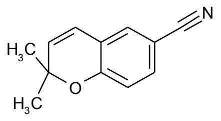	2,2-dimethyl-2H-chromene-6-carbonitrile	0 ± 0.07	80.4 ± 8.8	112.5 ± 14.6	108
488	217192-22-8	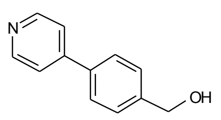	(4-pyrid-4-ylphenyl)methanol	-0.35 ± 0.07	89.5 ± 10.5	65.5 ± 17.5	133.3
489	217189-04-3	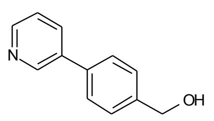	(4-pyrid-3-ylphenyl)methanol	-0.57 ± 0.08	82.7 ± 13.7	66.4 ± 18.8	131
490	98061-39-3	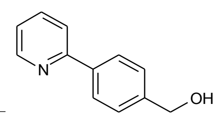	(4-pyrid-2-ylphenyl)methanol	-0.25 ± 0.08	77 ± 4.4	102.2 ± 13.5	117.6
491	85553-55-5	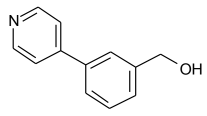	(3-pyrid-4-ylphenyl)methanol	-0.2 ± 0.07	**53.1 ± 10.8**	34.4 ± 13.8	66.8
492	4634-09-7	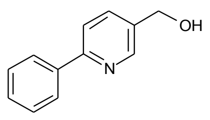	(6-phenyl-3-pyridinyl)methanol	-0.18 ± 0.08	59.3 ± 3.2	**78.6 ± 15.6**	81.3

Supplementary Table 1: Screening the Maybridge Rule of Three Fragment Library: TbINO1 differential scanning fluorimetry and cytotoxicity studies against bloodstream form *T.brucei* , HeLa and A549 cells.

493	175201-90-8	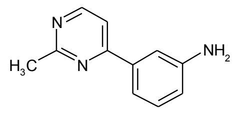	3-(2-methylpyrimidin-4-yl)aniline	0.15 ± 0.07	72.2 ± 9.4	**65.9 ± 17**	92.6
494	37128-24-8	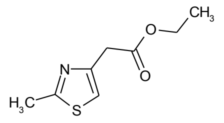	ethyl 2-(2-methyl-1,3-thiazol-4-yl)acetate	0.99 ± 0.08	107 ± 6.4	103 ± 17.9	119.5
495	859851-03-9	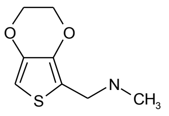	N-(2,3-dihydrothieno[3,4-b][1,4]dioxin-5-ylmethyl)-N-methylamine	-0.45 ± 0.07	28.2 ± 3.2	43.6 ± 17.7	96.9
496	55745-74-9	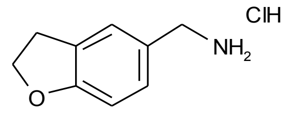	2,3-dihydrobenzo[b]furan-5-ylmethylamine hydrochloride	0.06 ± 0.08	75.4 ± 7.3	99.8 ± 15.5	96.7
497	4534-11-6	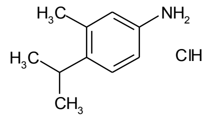	4-isopropyl-3-methylaniline hydrochloride	-0.21 ± 0.05	30.1 ± 9	**90.1 ± 2.1**	115.7
498	13600-49-2	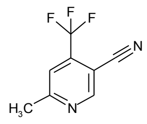	6-methyl-4-(trifluoromethyl)nicotinonitrile	-0.1 ± 0.09	**102.8 ± 15.5**	102.9 ± 18.5	107.7
499	312-73-2	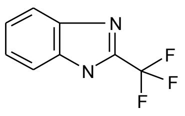	2-(trifluoromethyl)-1H-benzo[d]imidazole	-0.55 ± 0.07	**70.1 ± 5.1**	82 ± 14.1	116
500	654-70-6	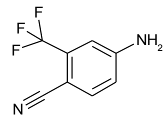	4-amino-2-(trifluoromethyl)benzonitrile	-1.28 ± 0.14	27 ± 2.3	**51 ± 10.6**	72.8
502	831-82-3	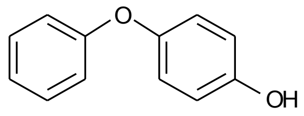	4-phenoxyphenol	-3.59 ± 0.1	30.7 ± 4.5	39.6 ± 16.6	53.1
503	10041-06-2	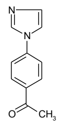	1-[4-(1H-imidazol-1-yl)phenyl]-1-ethanone	-0.33 ± 0.07	60.7 ± 7.5	69.7 ± 7.3	94.5
504	852180-75-7	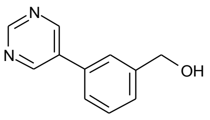	(3-pyrimidin-5-ylphenyl)methanol	-0.17 ± 0.07	87.8 ± 5.8	88.7 ± 9.9	88.2
505	198084-13-8	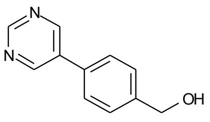	(4-pyrimidin-5-ylphenyl)methanol	-0.08 ± 0.08	**106.6 ± 3.1**	78.6 ± 20	85.3
506	86556-09-4	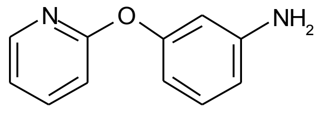	3-(pyridin-2-yloxy)aniline	0.23 ± 0.09	102.8 ± 5.4	99.2 ± 16.2	72.5
507	25194-67-6	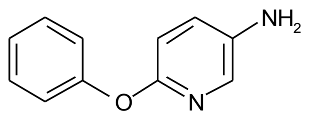	6-phenoxy-3-pyridinamine	-0.22 ± 0.07	49.5 ± 6	**103.3 ± 15.4**	71.8
508	3438-16-2	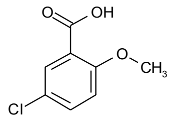	5-chloro-2-methoxybenzoic acid	0.07 ± 0.07	**92.5 ± 12**	**78.4 ± 17.3**	101.2
509	63968-85-4	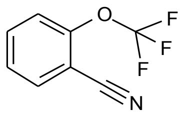	2-(trifluoromethoxy)benzonitrile	0.01 ± 0.08	114.1 ± 19.6	105.9 ± 16.9	110.4
510	635-80-3	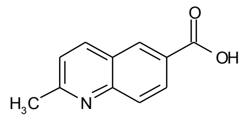	2-methyl-6-quinolinecarboxylic acid	0.12 ± 0.08	91.2 ± 16.1	116.6 ± 10.8	126
511	61471-45-2	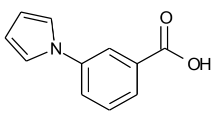	3-(1H-pyrrol-1-yl)benzoic acid	0.6 ± 0.08	46.2 ± 19.1	102.2 ± 12.3	73
512	22106-33-8	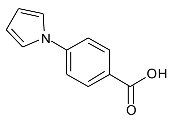	4-(1H-pyrrol-1-yl)benzoic acid	-4.93 ± 0.18	**42.1 ± 22.9**	97.5 ± 13.1	63.2
513	10333-68-3	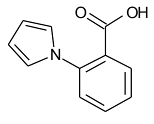	2-(1H-pyrrol-1-yl)benzoic acid	3.14 ± 0.09	**104.3 ± 9.6**	**98.5 ± 6.5**	64.4

Supplementary Table 1: Screening the Maybridge Rule of Three Fragment Library: TbINO1 differential scanning fluorimetry and cytotoxicity studies against bloodstream form *T.brucei* , HeLa and A549 cells.

514	27104-73-0	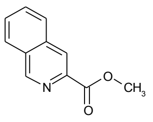	methyl isoquinoline-3-carboxylate	-0.37 ± 0.07	56.8 ± 18.2	79.5 ± 14.3	65.3
515	38896-30-9	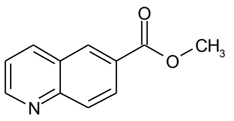	methyl quinoline-6-carboxylate	-0.1 ± 0.07	59.2 ± 1.4	**69.9 ± 8.1**	67.8
516	38084-44-5	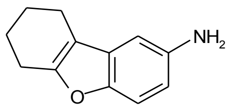	6,7,8,9-tetrahydrodibenzo[b,d]furan-2-amine	0.2 ± 0.07	**36.8 ± 1.1**	**108 ± 5.2**	68.2
517	780802-33-7	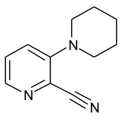	3-piperidin-1-ylpyridine-2-carbonitrile	0.16 ± 0.07	32.2 ± 8.8	51.2 ± 17.9	54.7
518	68104-63-2	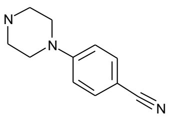	4-Piperazin-1-yl-benzonitrile	-0.26 ± 0.08	61.8 ± 2.1	92.1 ± 11	84.7
519	306937-35-9	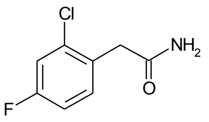	2-(2-chloro-4-fluorophenyl)acetamide	-0.3 ± 0.08	105.2 ± 15.1	98.3 ± 15.4	102.4
520	331942-47-3	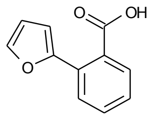	2-(2-furyl)benzoic acid	4.29 ± 0.07	35.4 ± 8.7	**73 ± 11.5**	123.9
521	111787-91-8	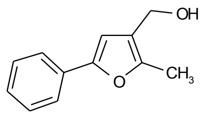	(2-methyl-5-phenyl-3-furyl)methanol	1.27 ± 0.07	35 ± 3.8	**41.5 ± 15**	57.1
522	183210-33-5	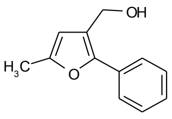	(5-methyl-2-phenyl-3-furyl)methanol	1.61 ± 0.06	45.4 ± 7.9	24.3 ± 17.4	27.1
523	153863-35-5	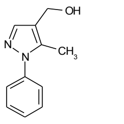	(5-methyl-1-phenyl-1H-pyrazol-4-yl)methanol	-0.01 ± 0.07	64.9 ± 10.9	75.9 ± 13.5	57.2
524	864068-97-3	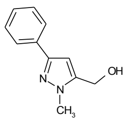	(1-methyl-3-phenyl-1H-pyrazol-5-yl)methanol	-0.34 ± 0.08	**85.9 ± 17.5**	88.2 ± 10.3	60.7
525	124344-98-5	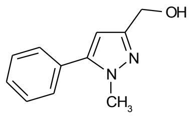	(1-methyl-5-phenyl-1H-pyrazol-3-yl)methanol	-0.44 ± 0.07	58 ± 13.5	64.8 ± 9.8	64.6
526	5376-10-3	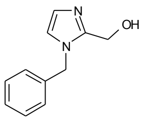	(1-benzyl-1H-imidazol-2-yl)methanol	-0.13 ± 0.07	84.5 ± 7.1	81 ± 13.3	58
527	151055-79-7	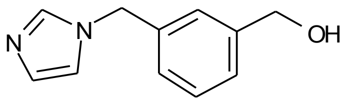	[3-(1H-imidazol-1-ylmethyl)phenyl]methanol	-0.31 ± 0.07	78.7 ± 10.9	80.7 ± 17.6	69.2
528	103573-92-8	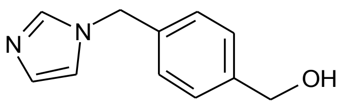	[4-(1H-imidazol-1-ylmethyl)phenyl]methanol	-0.2 ± 0.07	**78 ± 16**	90.8 ± 4.5	81.2
529	160388-55-6	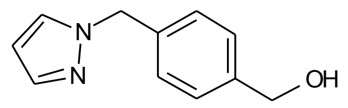	[4-(1H-pyrazol-1-ylmethyl)phenyl]methanol	-0.19 ± 0.08	**78.1 ± 10.3**	85 ± 5.2	94
530	499785-47-6	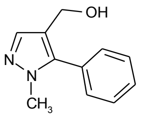	(1-methyl-5-phenyl-1H-pyrazol-4-yl)methanol	-0.19 ± 0.09	66.6 ± 15.6	87.4 ± 6.2	109.5
531	85953-29-3	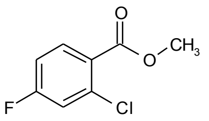	methyl 2-chloro-4-fluorobenzoate	0.25 ± 0.07	104 ± 17	84 ± 11.8	75.7
532	360-64-5	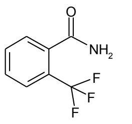	2-(trifluoromethyl)benzamide	0.43 ± 0.07	93.1 ± 13.1	88.3 ± 6.5	59.4
533	830-96-6	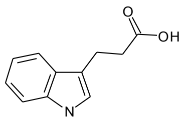	3-(1H-indol-3-yl)propanoic acid	0.73 ± 0.07	95.2 ± 14.2	94.9 ± 6	67.2

Supplementary Table 1: Screening the Maybridge Rule of Three Fragment Library: TbINO1 differential scanning fluorimetry and cytotoxicity studies against bloodstream form *T.brucei*, HeLa and A549 cells.

534	18718-79-1	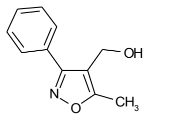	(5-methyl-3-phenyl-4-isoxazolyl)methanol	-0.6 ± 0.08	59.9 ± 18.3	78.8 ± 15.6	62.9
535	58161-35-6	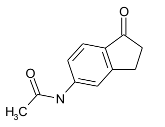	N1-(1-oxo-2,3-dihydro-1H-inden-5-yl)acetamide	0.21 ± 0.07	97.5 ± 6.6	87.3 ± 4.6	76.5
537	14844-73-6	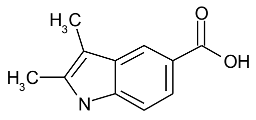	2,3-dimethyl-1H-indole-5-carboxylic acid	0.7 ± 0.08	**85.3 ± 0.3**	89.3 ± 2.4	74.5
539	455-24-3	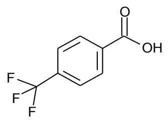	4-(trifluoromethyl)benzoic acid	-0.18 ± 0.08	80 ± 13.8	92.4 ± 8.2	103.6
540	51551-74-7	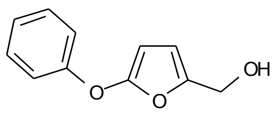	(5-phenoxy-2-furyl)methanol		29.9 ± 2.8	13.7 ± 6.5	57.7
541	175136-12-6	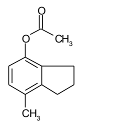	7-methyl-2,3-dihydro-1H-inden-4-yl acetate	0.01 ± 0.07	**45.5 ± 23.2**	79.8 ± 6.6	93.1
542	24197-95-3	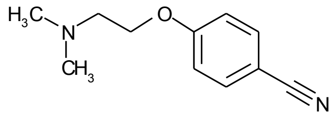	4-[2-(dimethylamino)ethoxy]benzonitrile	-0.18 ± 0.09	79.7 ± 7.3	91.6 ± 9.5	69.7
543	773872-97-2	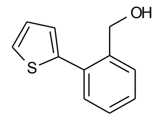	(2-thien-2-ylphenyl)methanol	-0.61 ± 0.07	30.9 ± 1.2	73.9 ± 3.7	56.5
544	81443-44-9	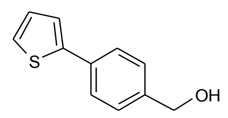	(4-thien-2-ylphenyl)methanol	0.16 ± 0.08	71.6 ± 7	74.7 ± 10.2	80
545	160278-20-6	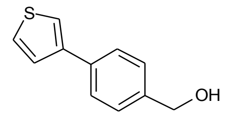	(4-thien-3-ylphenyl)methanol	-0.55 ± 0.1	**60.7 ± 9.2**	84.3 ± 13.2	87.2
546	89250-34-0	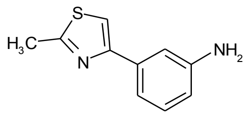	3-(2-methyl-1,3-thiazol-4-yl)aniline	-0.35 ± 0.08	52.8 ± 15.6	68.5 ± 8.2	61.9
547	25021-49-2	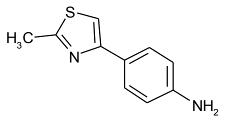	4-(2-methyl-1,3-thiazol-4-yl)phenylamine	0.12 ± 0.07	47.6 ± 17.4	74.3 ± 11	63.2
548	50541-93-0	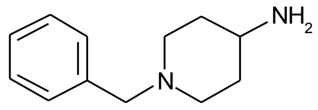	1-benzylpiperidin-4-amine	-0.23 ± 0.07	39.5 ± 10.8	39.5 ± 10	89.3
549	1013-22-5	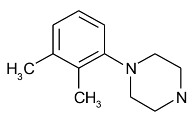	1-(2,3-dimethylphenyl)piperazine	-0.73 ± 0.07	25.8 ± 3.4	10.1 ± 5.7	13.6
550	91271-78-2	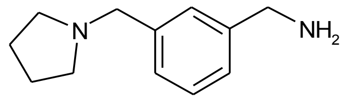	[3-(1-pyrrolidinylmethyl)phenyl]methanamine	0.03 ± 0.07	51.6 ± 9	47.8 ± 11.7	108.7
551	175137-11-8	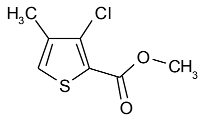	methyl 3-chloro-4-methylthiophene-2-carboxylate	-0.16 ± 0.07	108 ± 11.9	90.7 ± 10.3	104.9
552	212079-30-6	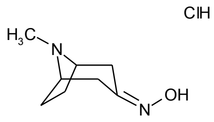	8-methyl-8-azabicyclo[3.2.1]octan-3-one oxime hydrochloride	0 ± 0.09	94.3 ± 14.4	103.5 ± 9.7	105.3
553	158063-66-2	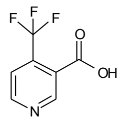	4-(trifluoromethyl)nicotinic acid	0.28 ± 0.07	102.6 ± 10.9	102.4 ± 12.4	102.6
554	208720-09-6	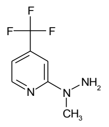	2-(1-methylhydrazino)-4-(trifluoromethyl)pyridine	0.15 ± 0.08	**96.3 ± 4.3**	**95.6 ± 16.7**	96.1
555	6624-49-3	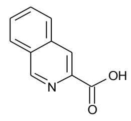	isoquinoline-3-carboxylic acid hydrate	0.5 ± 0.09	34.9 ± 11.7	92.1 ± 10.1	100.4

Supplementary Table 1: Screening the Maybridge Rule of Three Fragment Library: TbINO1 differential scanning fluorimetry and cytotoxicity studies against bloodstream form *T.brucei* , HeLa and A549 cells.

556	197899-76-6	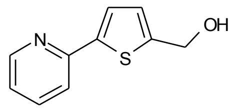	[5-(2-pyridinyl)-2-thienyl]methanol	0.19 ± 0.08	**128.4 ± 2.4**	70.2 ± 11	93.9
557	23780-13-4	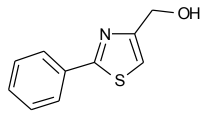	(2-phenyl-1,3-thiazol-4-yl)methanol	-0.18 ± 0.07	64.1 ± 11.9	58.1 ± 13.4	78.4
558	423768-57-4	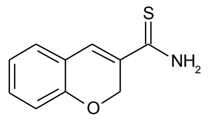	2H-chromene-3-carbothioamide	0.05 ± 0.09	**41.2 ± 13.3**	80.8 ± 8.8	98.1
559	91271-60-2	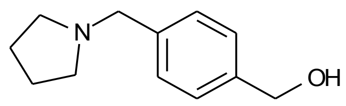	[4-(pyrrolidin-1-ylmethyl)phenyl]methanol	0.05 ± 0.08	82.9 ± 2.2	91.7 ± 15.8	110
560	58333-75-8	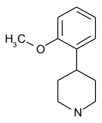	4-(2-methoxyphenyl)piperidine	-0.37 ± 0.08	25 ± 2.7	20.2 ± 2.8	63.7
561	180605-36-1	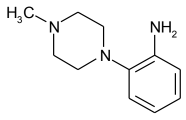	2-(4-methylpiperazin-1-yl)aniline	0.46 ± 0.07	72.8 ± 18.6	**74.3 ± 11.7**	101.2
562	16153-81-4	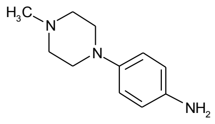	4-(4-methylpiperazino)aniline	-5.28 ± 0.14	15.6 ± 6.1	8.2 ± 6.5	17.5
563	1736-74-9	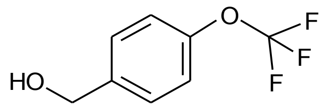	[4-(trifluoromethoxy)phenyl]methanol	0.01 ± 0.07	111.5 ± 10.3	94.5 ± 8.3	92.9
564	42327-95-7	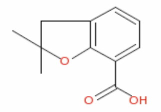	2,2-dimethyl-2,3-dihydro-1-benzofuran-7-carboxylic acid	0.35 ± 0.07	108.5 ± 16.1	91.1 ± 19	101.2
565	57634-55-6	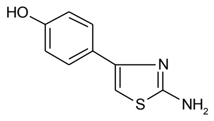	4-(2-amino-1,3-thiazol-4-yl)phenol	-4.02 ± 0.08	**48.7 ± 19.3**	**57.7 ± 17.7**	61.6
566	138745-99-0	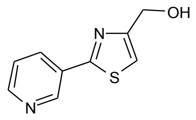	(2-pyridin-3-yl-1,3-thiazol-4-yl)methanol	0.29 ± 0.07	84.4 ± 17.3	88.4 ± 18.2	104
567	51013-67-3	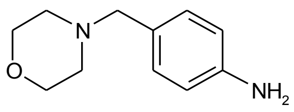	4-(morpholinomethyl)aniline	0.61 ± 0.05	**108.5 ± 0.2**	90.1 ± 15.9	102.7
568	888070-04-0	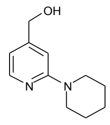	(2-piperidinopyrid-4-yl)methanol	0.03 ± 0.07	59.3 ± 16.3	**94.5 ± 18.7**	103
569	690631-99-3	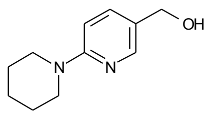	(Piperidino-3-pyridinyl)methanol	0.35 ± 0.07	31.1 ± 5.4	**89.2 ± 4.8**	106.4
570	95539-61-0	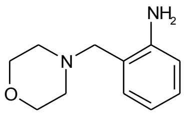	2-(morpholin-4-ylmethyl)aniline	0.35 ± 0.05	**123.3 ± 10.8**	93.2 ± 17.1	104.5
571	204078-48-8	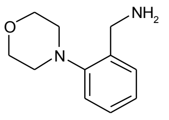	2-morpholinobenzylamine	0.06 ± 0.07	**65.8 ± 9.1**	95 ± 17.5	70.9
572	690632-84-9	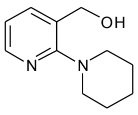	(2-piperidino-3-pyridinyl)methanol	1.25 ± 0.09	32.5 ± 9	**65.1 ± 5.7**	80.1
574	16718-11-9	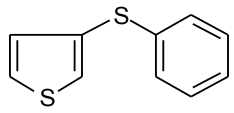	3-(phenylthio)thiophene	0.11 ± 0.07	112.3 ± 15.2	98.8 ± 18.5	93.9
575	133659-14-0	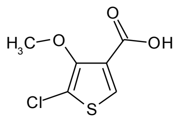	5-chloro-4-methoxythiophene-3-carboxylic acid	1.07 ± 0.08	84.1 ± 4.5	90.5 ± 15.4	91.2
576		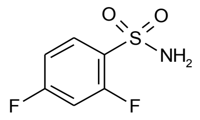	2,4-difluorobenzenesulfonamide	-6.33 ± 0.07	39.2 ± 8	10.7 ± 9.3	12.9

Supplementary Table 1: Screening the Maybridge Rule of Three Fragment Library: TbINO1 differential scanning fluorimetry and cytotoxicity studies against bloodstream form *T.brucei* , HeLa and A549 cells.

577	74772-17-1	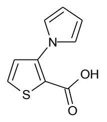	3-(1H-pyrrol-1-yl)thiophene-2-carboxylic acid	2.78 ± 0.08	**32.2 ± 4.1**	**103.2 ± 2.8**	90.4
578	280556-71-0	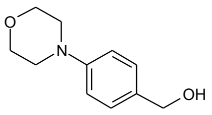	(4-morpholin-4-yl-phenyl)-methanol	0.17 ± 0.08	**93 ± 7.9**	**42.3 ± 9**	75.4
579	145127-38-4	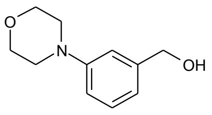	(3-morpholinophenyl)methanol	0.18 ± 0.08	69.7 ± 12.3	**100.1 ± 13.5**	100.8
580	465514-33-4	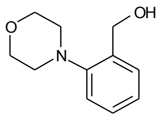	(2-morpholinophenyl)methanol	0.34 ± 0.06	**88.6 ± 0.9**	91.3 ± 16	122.7
581	37578-06-6	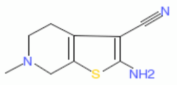	2-amino-6-methyl-4,5,6,7-tetrahydrothieno[2,3-c]pyridine-3-carbonitrile	1.39 ± 0.08	42.5 ± 18.5	**85.8 ± 12.8**	82.2
582	78583-81-0	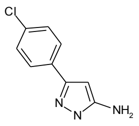	3-(4-chlorophenyl)-1H-pyrazol-5-amine	0.01 ± 0.07	100.5 ± 19.6	90.4 ± 12.2	68.6
583	17515-74-1	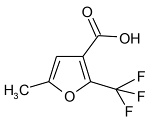	5-methyl-2-(trifluoromethyl)-3-furoic acid	0.06 ± 0.07	98.2 ± 11.7	86.1 ± 15.8	59.6
584	461-84-7	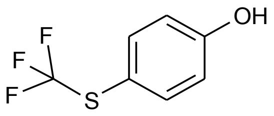	4-[(trifluoromethyl)thio]phenol	-0.92 ± 0.07	**94.2 ± 17.2**	97.5 ± 15.9	68.4
585	23795-02-0	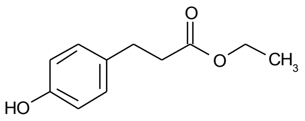	ethyl 3-(4-hydroxyphenyl)propanoate	-0.63 ± 0.08	**43.8 ± 15.9**	**63.1 ± 3.9**	61.7
586	879896-47-6	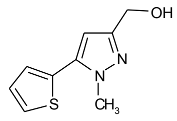	(1-methyl-5-thien-2-yl-1H-pyrazol-3-yl)methanol	-0.27 ± 0.07	71.4 ± 15.6	**82 ± 15.2**	63.4
587	879896-49-8	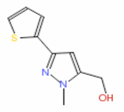	(1-methyl-3-thien-2-yl-1H-pyrazol-5-yl)methanol	0.36 ± 0.07	**61.3 ± 11.9**	**91.6 ± 8.8**	65.8
588	4942-47-6	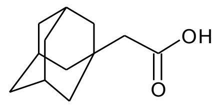	2-(1-adamantyl)acetic acid	-0.39 ± 0.07	48.1 ± 17.5	95.4 ± 14.1	85.4
589	1646-32-8	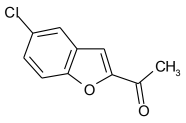	1-(5-chlorobenzo[b]furan-2-yl)ethan-1-one	-0.01 ± 0.08	**81.4 ± 11.7**	**95.8 ± 12.5**	99.5
590	36157-40-1	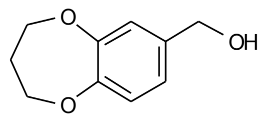	1-(2,5-dichloro-3-thienyl)ethan-1-one	0.3 ± 0.07	**105.6 ± 10.2**	97.7 ± 13.9	129.7
591	6476-32-0	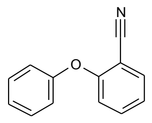	2-phenoxybenzonitrile	0.24 ± 0.06	**100.5 ± 1.9**	93 ± 2.5	58.3
592	690632-23-6	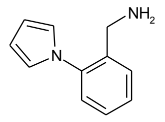	2,3-dihydro-1,4-benzodioxine-5-carbothioamide	0.18 ± 0.07	67.7 ± 17	54.3 ± 15.7	39.8
593	59115-26-3	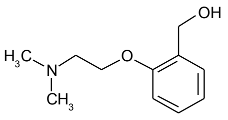	{2-[2-(dimethylamino)ethoxy]phenyl}methanol	0.13 ± 0.06	**103.6 ± 18.7**	77.9 ± 13.5	59.2
594	2199-47-5	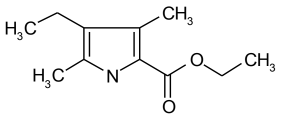	ethyl 4-ethyl-3,5-dimethyl-1H-pyrrole-2-carboxylate	0.17 ± 0.08	32.2 ± 12.3	**79.8 ± 6.6**	59.9
596	270260-33-8	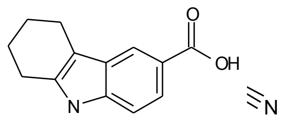	4-(pyridin-2-yloxy)benzonitrile	0.68 ± 0.07	**89.3 ± 9**	**97.7 ± 11**	72.1
597	42711-75-1	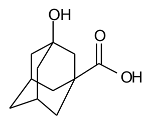	3-hydroxyadamantane-1-carboxylic acid	0.29 ± 0.06	**105.8 ± 11**	75.2 ± 12.6	79.4

Supplementary Table 1: Screening the Maybridge Rule of Three Fragment Library: TbINO1 differential scanning fluorimetry and cytotoxicity studies against bloodstream form *T.brucei* , HeLa and A549 cells.

598	3515-30-8	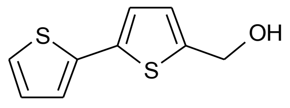	2,2'-bithien-5-ylmethanol	1.72 ± 0.06	55.3 ± 15.6	**78 ± 11.6**	86.3
599	321309-35-7	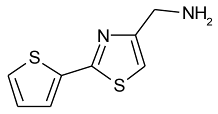	[2-(2-thienyl)-1,3-thiazol-4-yl]methylamine	0.41 ± 0.06	**35.2 ± 15.5**	**71.9 ± 7.1**	98.4
600	6537-46-8	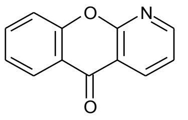	5H-chromeno[2,3-b]pyridin-5-one	0.62 ± 0.05	**80.8 ± 9.9**	78.4 ± 11	126
601	2835-78-1	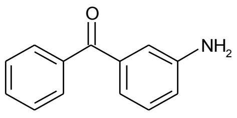	(3-aminophenyl)(phenyl)methanone	0.21 ± 0.06	97.7 ± 12.1	89.2 ± 6.6	63.8
602	131947-13-2	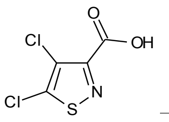	4,5-dichloroisothiazole-3-carboxylic acid	0.1 ± 0.07	31.7 ± 9.1	83.2 ± 12.5	68.2
603	5221-44-3	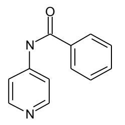	N1-(4-pyridyl)benzamide	0.32 ± 0.07	82.8 ± 5	79 ± 15.1	57.3
604	10297-73-1	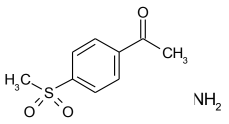	1-[4-(methylsulfonyl)phenyl]ethan-1-one	0.18 ± 0.06	**108.6 ± 11.7**	83.1 ± 19.6	63.9
605	852180-67-7	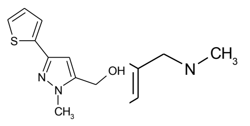	N-methyl-N-(3-pyridin-4-ylbenzyl)amine	0.05 ± 0.07	37.4 ± 13.7	21.3 ± 13.8	36.2
606	852180-72-4	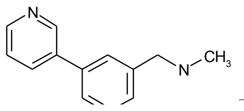	N-methyl-N-(3-pyridin-3-ylbenzyl)amine	0.07 ± 0.08	43.2 ± 12.9	**70.8 ± 5.8**	69.8
607	869901-08-6	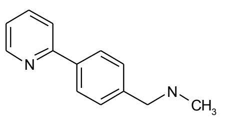	N-methyl-N-(4-pyridin-2-ylbenzyl)amine	0.02 ± 0.06	32.8 ± 7.4	57.7 ± 17.1	71.5
608	306934-93-0	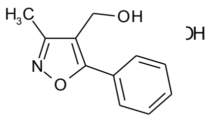	(5-chloro-1-benzothiophen-3-yl)methanol	-1.33 ± 0.1	**99 ± 26.1**	**78.3 ± 1**	63.8
609	13735-12-1	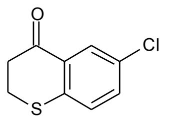	6-chloro-3,4-dihydro-2H-1-benzothiin-4-one	2.49 ± 0.04	**86.3 ± 6.6**	**74.5 ± 13.7**	104.8
610	29051-44-3	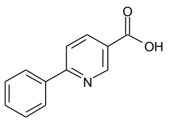	6-phenylnicotinic acid	0.46 ± 0.06	78.4 ± 13.4	82.3 ± 8.2	125.4
611	4467-07-6	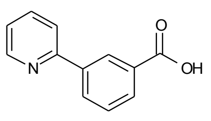	3-pyrid-2-ylbenzoic acid	0.19 ± 0.07	98.5 ± 13.5	87 ± 12.6	73.9
612	4385-75-5	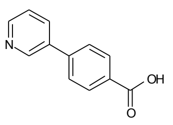	4-pyrid-3-ylbenzoic acid	-0.97 ± 0.06	40.1 ± 12.4	**77.2 ± 5.7**	74.2
613	134363-45-4	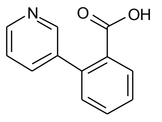	2-pyrid-3-ylbenzoic acid	0.14 ± 0.07	80.1 ± 7	80 ± 5.6	59.8
614	4385-78-8	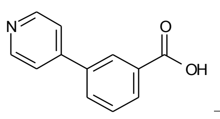	3-pyrid-4-ylbenzoic acid	0.46 ± 0.12	41.6 ± 12.5	81.2 ± 15.9	66.3
615	10177-12-5	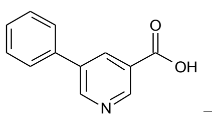	5-phenylnicotinic acid	1.28 ± 0.09	77.6 ± 10.5	80.4 ± 11.7	75.6
616	93189-16-3	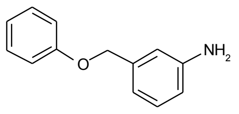	3-(phenoxymethyl)aniline	-1.45 ± 0.08	35.4 ± 10.2	80.5 ± 17.9	57.5
617	4815-24-1	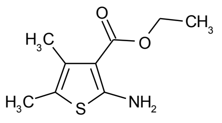	ethyl 2-amino-4,5-dimethylthiophene-3-carboxylate	1.47 ± 0.07	**100.4 ± 13.8**	69.1 ± 9.3	75.2
618	910036-90-7	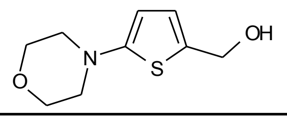	(5-morpholinothien-2-yl)methanol	1.12 ± 0.11	29.7 ± 4.6	70.4 ± 13.2	76.3

Supplementary Table 1: Screening the Maybridge Rule of Three Fragment Library: TbINO1 differential scanning fluorimetry and cytotoxicity studies against bloodstream form *T.brucei* , HeLa and A549 cells.

619	19228-91-2	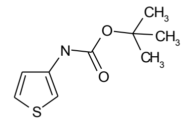	tert-butyl N-(3-thienyl)carbamate	0.21 ± 0.07	60.5 ± 3.8	80 ± 17.5	74.9
620	56267-50-6	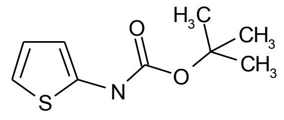	tert-butyl N-(2-thienyl)carbamate	2.08 ± 0.1	40.1 ± 17.6	71.3 ± 13.6	109.4
621	13807-84-6	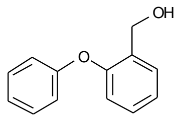	(2-phenoxyphenyl)methanol	-0.77 ± 0.08	45 ± 4.4	74.9 ± 14.8	73.7
622	2215-78-3	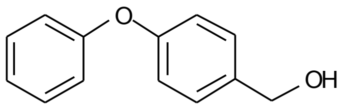	(4-phenoxyphenyl)methanol	-0.33 ± 0.07	84 ± 5.4	78.3 ± 15.4	70.7
623	342405-27-0	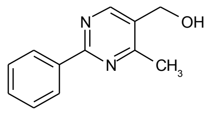	(4-methyl-2-phenyl-5-pyrimidinyl)methanol	-0.05 ± 0.07	61.8 ± 14.4	**66.5 ± 11.6**	85.4
624	24016-03-3	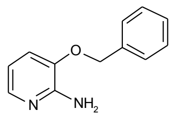	3-(benzyloxy)pyridin-2-amine	-0.32 ± 0.08	**70.4 ± 12.1**	**72.9 ± 14.8**	82.7
625	127406-79-5	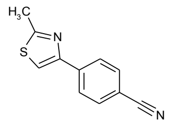	4-(2-methyl-1,3-thiazol-4-yl)benzonitrile	1.06 ± 0.07	83.5 ± 5.3	**74.1 ± 7.1**	79.4
626	29198-41-2	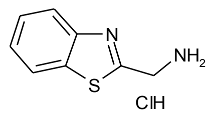	1,3-benzothiazol-2-ylmethylamine hydrochloride	2.03 ± 0.08	52.9 ± 13.9	**95.1 ± 15.5**	85.7
628	10333-67-2	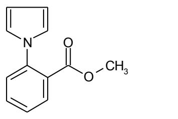	methyl 2-(1H-pyrrol-1-yl)benzoate	0.84 ± 0.08	**35.1 ± 9.3**	70.4 ± 14	85.1
629	194017-70-4	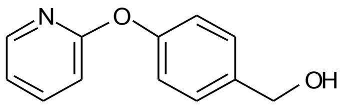	[4-(pyrid-2-yloxy)phenyl]methanol	-0.15 ± 0.07	82 ± 5.4	84.1 ± 9.9	96.1
630	869901-22-4	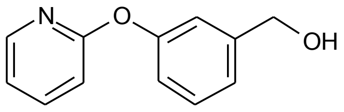	[3-(pyrid-2-yloxy)phenyl]methanol	-0.36 ± 0.07	**81.4 ± 5.7**	80 ± 1	98.3
631	864068-98-4	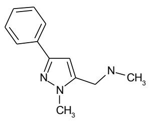	N-methyl-N-[(1-methyl-3-phenyl-1H-pyrazol-5-yl)methyl]amine	0.5 ± 0.08	31 ± 6.5	66.2 ± 16.3	100.2
632	17841-68-8	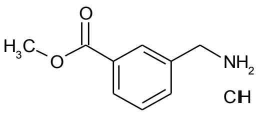	3-Aminomethylbenzoic acid methyl ester hydrochloride	0.22 ± 0.07	57.9 ± 14.5	44.8 ± 10.2	107.3
633	35370-95-7	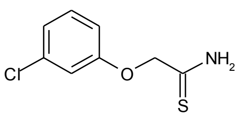	2-(3-chlorophenoxy)ethanethioamide	-0.45 ± 0.14	**86.5 ± 6.5**	46.9 ± 11.1	90.3
634	680593-95-7	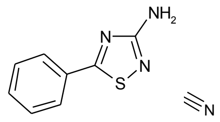	2-{[5-(trifluoromethyl)-2-pyridyl]oxy}acetonitrile	0.24 ± 0.09	**80.4 ± 15**	**73.3 ± 1.6**	87.2
635	64354-50-3	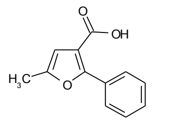	5-methyl-2-phenyl-3-furoic acid	0.38 ± 0.04	40.5 ± 7.4	17.7 ± 16.2	90.1
636	58789-53-0	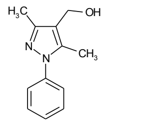	(3,5-dimethyl-1-phenyl-1H-pyrazol-4-yl)methanol	-0.18 ± 0.07	70.4 ± 15.2	73 ± 13.4	94.9
637	857283-57-9	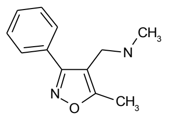	N-methyl-N-[(5-methyl-3-phenylisoxazol-4-yl)methyl]amine	0.02 ± 0.07	66.1 ± 6.7	79.8 ± 7.5	112.6
638	15190-10-0	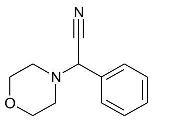	2-morpholino-2-phenylacetonitrile	-0.29 ± 0.09	**94.8 ± 10**	80.5 ± 8.2	89.8
639	4335-28-8	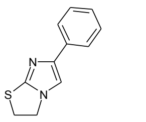	6-phenyl-2,3-dihydroimidazo[2,1-b][1,3]thiazole	-1.69 ± 0.03	31.1 ± 5.8	61 ± 9.8	109.1

Supplementary Table 1: Screening the Maybridge Rule of Three Fragment Library: TbINO1 differential scanning fluorimetry and cytotoxicity studies against bloodstream form *T.brucei* , HeLa and A549 cells.

641	3405-88-7	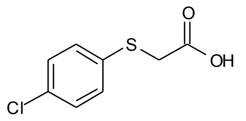	2-[(4-chlorophenyl)thio]acetic acid	-0.53 ± 0.07	**129.4 ± 24.6**	105.2 ± 3.7	118.7
643	103788-65-4	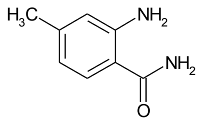	2-(5-methyl-2-phenyl-1,3-oxazol-4-yl)ethan-1-ol	-0.74 ± 0.07	28.1 ± 8.8	45.5 ± 7.7	104.4
644	88611-67-0	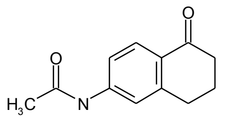	N1-(5-oxo-5,6,7,8-tetrahydronaphthalen-2-yl)acetamide	-0.13 ± 0.08	**104.2 ± 9.2**	99.2 ± 7.5	93.5
645	884507-34-0	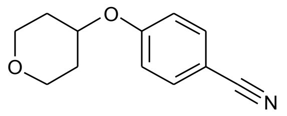	4-(tetrahydropyran-4-yloxy)benzonitrile	0.18 ± 0.05	**121 ± 12.5**	77.4 ± 15.8	101.5
646	852180-66-6	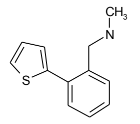	N-methyl-N-(2-thien-2-ylbenzyl)amine	0.1 ± 0.05	27.9 ± 11	36.5 ± 9.5	66.9
648	29886-64-4	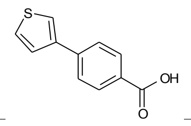	4-(3-thienyl)benzoic acid	0.25 ± 0.08	71.2 ± 17.9	105.3 ± 8.2	149.3
649	19163-24-7	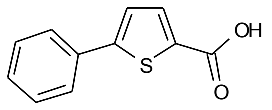	5-phenylthiophene-2-carboxylic acid	-2.8 ± 0.08	**71.2 ± 14.3**	**121.8 ± 18.7**	140.7
650	20608-89-3	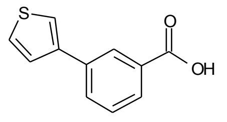	3-thien-3-ylbenzoic acid	-0.96 ± 0.07	**30.5 ± 9**	113.5 ± 15.9	165.2
651	3430-40-8	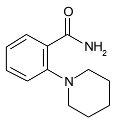	2-piperidinobenzamide	-1.33 ± 0.07	31.6 ± 9.7	82.4 ± 5.4	93.1
653	892501-89-2	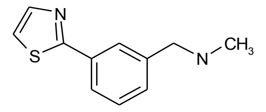	N-methyl-3-(1,3-thiazol-2-yl)benzylamine	-0.96 ± 0.69	26.6 ± 6.5	**93.3 ± 2.7**	99.6
654	886851-31-6	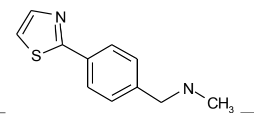	N-methyl-4-(1,3-thiazol-2-yl)benzylamine	-0.49 ± 0.06	28.2 ± 6.4	66.2 ± 16.9	113.7
655	91271-80-6	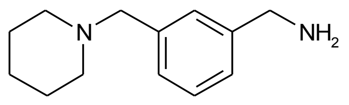	3-piperidin-1-ylmethyl benzylamine	-0.13 ± 0.06	59.2 ± 10.9	73.7 ± 15.9	126.1
656	859850-65-0	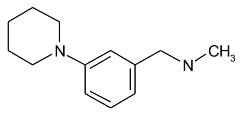	N-methyl-N-(3-piperidin-1-ylbenzyl)amine	-0.36 ± 0.05	28.2 ± 9.7	27.4 ± 13.3	103.5
657	852180-55-3	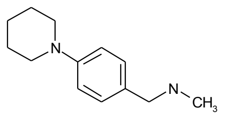	N-methyl-N-(4-piperidin-1-ylbenzyl)amine	-0.79 ± 0.06	27.3 ± 8.2	40.9 ± 12	77.4
658	91271-81-7	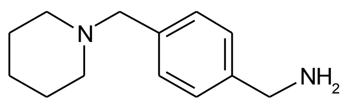	[4-(piperidinomethyl)phenyl]methylamine	-0.23 ± 0.08	46.6 ± 16.8	**22.8 ± 5.5**	123.5
659	5807-30-7	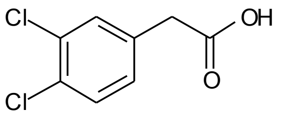	2-(3,4-dichlorophenyl)acetic acid	-0.42 ± 0.07	**36.2 ± 19.3**	74 ± 17.1	106.4
660	716-03-0	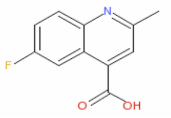	6-fluoro-2-methyl-4-quinolinecarboxylic acid	0.5 ± 0.06	**115.1 ± 0.7**	103 ± 3.9	146.9
661	119082-97-2	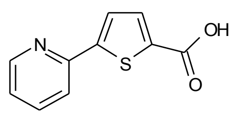	5-(2-pyridyl)thiophene-2-carboxylic acid	0.91 ± 0.07	89.3 ± 17.6	95.3 ± 3.5	94.1
662	266369-49-7	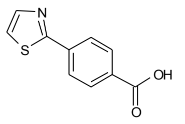	4-(1,3-thiazol-2-yl)benzoic acid	0.36 ± 0.06	**116.4 ± 24**	102 ± 7.2	100.5
663	77940-94-4	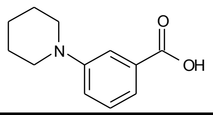	3-piperidinobenzoic acid	1.23 ± 0.07	**78.6 ± 22.6**	**97.9 ± 1.5**	104.1

Supplementary Table 1: Screening the Maybridge Rule of Three Fragment Library: TbINO1 differential scanning fluorimetry and cytotoxicity studies against bloodstream form *T.brucei*, HeLa and A549 cells.

664	4721-98-6	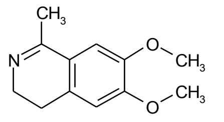	6,7-dimethoxy-1-methyl-3,4-dihydroisoquinoline	-0.27 ± 0.07	48.8 ± 12	75.4 ± 2.2	77.7
665	159691-25-5	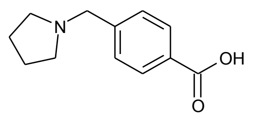	4-(pyrrolidin-1-ylmethyl)benzoic acid	-0.14 ± 0.07	**79.6 ± 19.9**	90.3 ± 6.3	103.9
666	857284-12-9	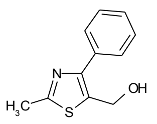	(2-methyl-4-phenyl-1,3-thiazol-5-yl)methanol	0.08 ± 0.05	**85.9 ± 17.4**	95.3 ± 5	113.4
667	857283-96-6	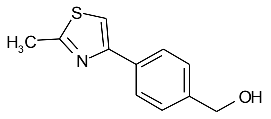	[4-(2-methyl-1,3-thiazol-4-yl)phenyl]methanol	0.4 ± 0.07	**41.7 ± 12.6**	76.7 ± 4.3	103.8
668	884507-30-6	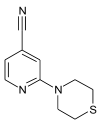	2-thiomorpholinoisonicotinonitrile	1.06 ± 0.07	**106.2 ± 23.6**	80.3 ± 19.6	98.5
669	91271-62-4	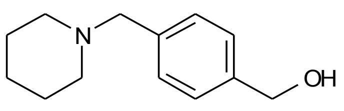	[4-(piperidin-1-ylmethyl)phenyl]methanol	-0.3 ± 0.07	72.2 ± 11.3	93.2 ± 12.6	121
670	19577-84-5	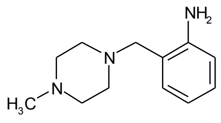	2-[(4-methylpiperazin-1-yl)methyl]aniline	0.05 ± 0.07	**52.6 ± 17.6**	94.6 ± 8.5	120.8
671	879896-58-9	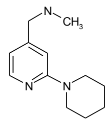	N-methyl-N-[(2-piperidin-1-ylpyridin-4-yl)methyl]amine	-0.49 ± 0.06	27.3 ± 6.8	54.8 ± 9.2	82.7
672	7238-62-2	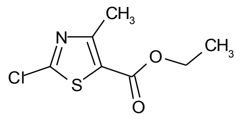	ethyl 2-chloro-4-methyl-1,3-thiazole-5-carboxylate	-0.22 ± 0.07	87.2 ± 12.5	89.6 ± 12.5	60.5

[a] Arbitrary library number

[b] CAS numbers are unique identifiers assigned by the "Chemical Abstracts Service" to describe every chemical described in open access scientific literature.

[c] Tm shift in °C, observed for TbINO1 in the presence of compound (1mM), value is mean ± SD from the Boltzman curve fitting, see Experimental for details.

[d] Cytotoxicity studies, see Experimental for details, values are percentage of controls in the absence of compound, either mean ± SD (n=3) or mean ± SE (n=2), the latter being in bold.

[e] Cytotoxicity studies, see Experimental for details, values are percentage of controls in the absence of compound, (n=1).

[f] TbINO1 Tm in presence of DMSO (true control), shows slight plate to plate variation, value is mean ± SD. The positive controls, 5 mM glucose-6-phosphate and 4 mM 2-deoxy-glucose-6-phosphate had Tm values (mean ± SD) of 0.70 ± 0.28°C and 2.84 ± 0.47°C, respectively.
